# Exercise prescription for improving chronic low back pain in adults: a network meta-analysis

**DOI:** 10.3389/fpubh.2025.1512450

**Published:** 2025-05-30

**Authors:** Ke Zhao, Ping Zhang, Hua Li, Li Li

**Affiliations:** ^1^Graduate School of Harbin Sport University, Harbin, Heilongjiang, China; ^2^College of Physical Education, Zhangjiakou University, Zhangjiakou, Hebei, China

**Keywords:** chronic low back pain, exercise prescription, network meta-analysis, Tai Chi, intervention effectiveness

## Abstract

**Objective:**

This study aims to investigate the impact of various combinations of exercise prescription variables—namely type, duration, frequency, and period—on improving chronic low back pain (CLBP) in adults. The goal is to provide evidence to inform the development of exercise prescriptions for CLBP interventions.

**Methods:**

Data sources were obtained from EBSCO, PubMed, Web of Science, Embase, and Cochrane, with the search conducted up to June 30, 2024. Two independent reviewers screened the literature, extracted data, and assessed the risk of bias in the included studies. A network meta-analysis (NMA) was performed using Stata 17.0 software, and the surface under the cumulative ranking (SUCRA) was utilized to rank the effectiveness of the exercise prescription variables.

**Results:**

Significant effects were observed for durations of 15–30 min [SMD = −1.62, 95% CI (−2.32, −0.92)] and ≥60 min [SMD = −0.81, 95% CI (−1.58, −0.03)] when compared to the control group. Intervention periods of 4 weeks [SMD = −1.82, 95% CI (−3.37, −0.28)], 12 weeks [SMD = −1.18, 95% CI (−1.85, −0.51)], and ≥16 weeks [SMD = −2.75, 95% CI (−4.26, −1.24)] also resulted in significantly better outcomes compared to the control group. The intervention effect for durations of ≥16 weeks was significantly greater than that for 12 weeks [SMD = −2.17, 95% CI (−3.58, −0.47)] and 6 weeks [SMD = −2.18, 95% CI (−3.85, −0.45)]. A frequency of three sessions per week [SMD = −1.44, 95% CI (−2.09, −0.78)] demonstrated significantly superior outcomes compared to the control group. An intervention duration of 15 to 30 min (SUCRA = 94.6), three sessions per week (SUCRA = 87), an intervention period of ≥16 weeks (SUCRA = 95.4), and Tai Chi exercise (SUCRA = 77.4) may be the most effective approaches for improving chronic low back pain in adults.

**Conclusion:**

Tai Chi exercise, lasting 15 to 30 min per session, performed three times a week over an intervention period of at least 16 weeks, may represent the most effective intervention for alleviating chronic low back pain in adults. However, due to the limited number of studies included, further research is necessary to provide stronger evidence.

## 1 Introduction

Chronic low back pain is characterized by persistent discomfort in the lower back, lumbosacral, and sacroiliac regions lasting more than three months. This pain may radiate to the buttocks or lower limbs, with or without accompanying radiating pain (1, 2). The condition significantly affects both the physical and mental health of patients, limiting daily activities, reducing sleep quality, and potentially leading to a substantial psychological burden, often manifested as anxiety, depression, and other emotional disorders (3). The high prevalence of chronic low back pain increases the strain on healthcare resources, as patients frequently seek medical attention and incur considerable treatment costs (4, 5). Furthermore, its effect on work performance can result in indirect economic losses, ultimately placing significant economic and social pressures on individuals, families, and society at large (6–8).

In recent years, the continuous advancement of health science research has led to an increased focus on non-pharmacological treatment approaches. Compared to traditional pharmacological treatments, exercise interventions, as a form of non-pharmacological therapy, present potential advantages, including reduced drug dependency, lower risk of side effects, and enhanced overall health (9). Numerous studies have demonstrated that appropriate exercise can strengthen lumbar muscles, improve spinal stability, and promote local blood circulation, effectively alleviating symptoms of chronic low back pain (10, 11). These exercise interventions encompass core stability training, yoga, Pilates, and Tai Chi, among others (12–15). While these studies provide robust evidence for the effectiveness of exercise in managing chronic low back pain (16–18), the optimal combination of exercise prescription—specifically, type, duration, frequency, and period—remains unclear for achieving the most effective intervention in adults with chronic low back pain.

The advantage of network meta-analysis lies in its ability to indirectly compare the effects of various interventions through their effects as intermediaries, even in the absence of direct comparison evidence. This approach overcomes the limitations of traditional meta-analysis, which can only handle direct comparisons between two interventions, thereby enhancing the precision of the analysis (19). Currently, treatment protocols for chronic low back pain in adults still require further exploration, particularly in determining the most effective types of exercise interventions, including their duration, frequency, and period, to develop more targeted exercise prescriptions (20–22). Therefore, this study aims to conduct a network meta-analysis of randomized controlled trials on exercise interventions for chronic low back pain in adults, providing specific recommendations for alleviating low back pain symptoms.

## 2 Data and methods

This systematic review was conducted by the Preferred Reporting Items for Systematic Reviews and Meta-Analyses (PRISMA) statement (23) and prospectively registered on the PROSPERO platform, with registration number CRD42024595808 (24). Additionally, the study protocol has been published, and the results are reported by the PRISMA Extension for Network Meta-Analysis (PRISMA-NMA) guidelines (refer to the PRISMA-NMA checklist in Supplementary material) (25).

### 2.1 Literature search strategy

The following databases were searched: EBSCO, PubMed, Web of Science, Embase, and Cochrane databases. The search was conducted until June 30, 2024, for each respective database. The search terms used included: ① exercise, strength training, physical exercise, physical activity, Pilates, sports, fitness, functional training, cardio training, yoga, exercise therapy; ② adult, mature, grown-up, adulthood, middle-aged, older adult, senior, full-grown, professional, independent, mature individual, established person; ③ CLBP, chronic back pain, lumbar pain, low backache, chronic lumbago, persistent backache, recurring low back pain, chronic lumbar dysfunction, persistent lumbosacral pain, musculoskeletal pain, disc degeneration, sacroiliac joint dysfunction, RCT, experiment, trial. Boolean operator “AND” was used to connect the three sets of terms. Furthermore, relevant studies were identified by tracing articles from published systematic reviews and meta-analyses to ensure comprehensive coverage of the literature. For instance, the search strategy for Web of Science was as follows: TS = (exercise OR “Strength Training” OR “physical exercise” OR “physical activity” OR Pilates OR sports OR fitness OR “Functional Training” OR “Cardio Training” OR Yoga OR “Exercise Therapy”) AND TS = (Adult OR Mature OR “Grown-up” OR Adulthood OR “Middle-aged” OR Older adult OR Senior OR “Full-grown” OR Professional OR Independent OR “Mature Individual” OR “Established Person”) AND TI = (CLBP OR “Chronic Back Pain” OR “Lumbar Pain” OR “Low Backache” OR “Chronic Lumbago” OR “Persistent Backache” OR “Recurring Low Back Pain” OR “Chronic Lumbar Dysfunction” OR “Persistent Lumbosacral Pain” OR “Musculoskeletal Pain” OR “Disc Degeneration” OR “Sacroiliac Joint Dysfunction” OR RCT OR experiment OR trial).

### 2.2 Inclusion and exclusion criteria

The inclusion and exclusion criteria were defined according to the PICOS framework (26). Duplicate records from the retrieved literature were eliminated using EndNote 20 software (27). Two independent reviewers performed the screening process in a double-blind manner, adhering to the inclusion and exclusion criteria. The reviewers first conducted a preliminary reading of titles and abstracts to identify potentially eligible studies. Full-text articles of studies that appeared to meet the inclusion criteria were then retrieved and further assessed, with final inclusion decisions made according to the criteria. The inclusion criteria were as follows: (1) Population: Adults aged 18 years and older with chronic low back pain (CLBP) lasting more than 3 months, specifically non-specific chronic low back pain. (2) Interventions: Studies including any form of exercise intervention were eligible. No mandatory requirements were specified for the type, duration, or frequency of exercise, but the intervention period was required to last at least 4 weeks. (3) Control: Studies including a control group receiving usual care, such as routine daily activities, health education, or conventional nursing. (4) Outcomes: The Visual Analog Scale (VAS) was used to measure treatment outcomes. (5) Study Design: Randomized controlled trials (RCTs). The exclusion criteria were as follows: (1) Cross-sectional studies, case–control studies, and other descriptive research designs. (2) Reviews, abstracts, letters, and commentaries lacking a clear description of the study design. (3) Articles with incomplete data that could not be obtained from alternative sources. (4) Studies involving structural issues, such as osteoporosis, scoliosis, fractures, or inflammatory conditions causing low back pain.

### 2.3 Data extraction

Two members of the research team, both trained in evidence-based methodology and possessing extensive experience in the field of chronic low back pain in adults, independently conducted the literature screening and data extraction. In cases where discrepancies arose during the screening or extraction process, a third team member, possessing significant expertise in the treatment of chronic low back pain in adults, was consulted to provide guidance. The final decision was made through discussion and consensus. The data extraction primarily focused on the following key information: first author’s name, publication year, the country where the study was conducted, sample size of adult participants, type of intervention implemented, duration of the intervention, frequency of the intervention, intervention period, and primary outcome measures used to assess the effectiveness of the intervention.

### 2.4 Bias risk assessment

In this study, an independent bias risk assessment was conducted for all included studies using the Cochrane Risk of Bias Tool, by the PROSPERO registration statement (28). This assessment framework comprehensively addresses seven key dimensions: the validity of the randomization method, the implementation of blinding for trial participants and personnel, the blinding status of outcome assessors, the concealment of the allocation process, the completeness and accuracy of outcome data, the presence of selective reporting of outcomes, and the potential for other biases. The quality risk of each study was categorized into three levels: low risk, high risk, and unclear risk. In the case of discrepancies in the bias risk assessment, the reviewers reached a consensus through discussion. If a consensus could not be reached, the corresponding author made the final decision based on their judgment, considering the opinions of the majority of reviewers.

### 2.5 Statistical methods

In this study, global inconsistency was tested, and the test result showed a *p*-value of 0.520, which is greater than 0.05, indicating good global consistency. Further testing for consistency within each closed loop revealed that the inconsistency factor was closer to zero, suggesting better consistency. The results showed that the lower limits of the inconsistency factors included zero, suggesting no significant inconsistency between direct and indirect comparisons. Subsequently, the analysis was performed using Stata 17.0 software (29). Given that the outcome measures were continuous data and that different studies used varying assessment tools and measurement units, the standardized mean difference (SMD) was selected as the effect size measure to ensure consistency and comparability across studies, allowing precise calculation of the combined effect size. Network meta-analysis is a method that integrates both direct and indirect evidence, allowing comparisons of more than two interventions simultaneously. In the network plot, each node represents an intervention, with the size of the node reflecting the sample size, while the thickness of the connecting lines indicates the number of studies included. To further compare the efficacy of different interventions, the SUCRA (Surface Under the Cumulative Ranking Curve) method was used to rank the interventions. SUCRA is a statistical method based on network meta-analysis, employed to quantify the relative efficacy of each intervention among all possible interventions. The value ranges from 0 to 100, with higher values indicating better relative efficacy in improving chronic non-specific low back pain. Specifically, the standardized mean difference (SMD) and its 95% confidence interval were first calculated for each intervention, followed by the estimation of the cumulative ranking curves of each intervention through the network meta-analysis model, and ultimately ranking all interventions based on the SUCRA values. A higher SUCRA value indicates a greater likelihood that the intervention is the best treatment (30).

### 2.6 Evidence certainty assessment

In this study, the GRADE system assessment tool was used to evaluate the quality of evidence for all outcome indicators, and the results showed that there were 2 high-level evidence, 17 moderate-level evidence, 5 low-level evidence, and 2 very low-level evidence (see Table 1). Among the downgrading factors, limitations were the main downgrading factor, 24 were downgraded because of limitations, most of the literature only mentioned randomization without describing the method of generating random numbers, most of the literature did not use blinding and allocation concealment, and only a few of the literature described the process of single or double blinding and allocation concealment.

**Table 1:** Evaluation of the quality of evidence in the included literature.

Author & year	Limitations	Inconsistency	Indirectness	Imprecision	Publication bias	Level of evidence
Akhtar et al. (14)	0	0	0	0	0	High
Arampatzis et al. (56)	−1	0	0	0	0	Moderate
Gladwell et al. (17)	−1	0	0	0	0	Moderate
Bae et al. (79)	−1	0	0	0	0	Moderate
Oh et al. (80)	−1	0	0	−1	0	Low
Cho et al. (81)	−1	0	0	0	0	Moderate
Cho et al. (82)	−1	0	−1	0	0	Low
Hwangbo et al. (83)	−1	0	−1	0	0	Low
Liu et al. (41)	−1	0	0	0	0	Moderate
Williams et al. (84)	−1	−1	0	0	0	Low
Kumar (12)	0	0	0	0	0	High
Lee and Kang (85)	−1	0	0	0	0	Moderate
Michaelson et al. (86)	−1	−1	−1	0	0	Very Low
Noormohammadpour et al. (87)	−1	−1	0	0	0	Low
Roh et al. (88)	−1	0	0	0	0	Moderate
Shamsi et al. (15)	−1	0	0	0	0	Moderate
Tang et al. (18)	−1	0	0	0	0	Moderate
Tekur et al. (89)	−1	0	0	0	0	Moderate
Teut et al. (30)	−1	−1	−1	0	0	Very Low
Ulger et al. (67)	−1	0	0	0	0	Moderate
Ulger et al. (90)	−1	0	0	0	0	Moderate
Williams et al. (91)	−1	0	0	0	0	Moderate
Yoo and Lee et al. (57)	−1	0	0	0	0	Moderate
Zhang et al. (92)	−1	0	0	0	0	Moderate
Koumantakis et al. (13)	−1	0	0	0	0	Moderate
You et al. (16)	−1	0	0	0	0	Moderate

## 3 Results

### 3.1 Literature search results

**Figure 1:**
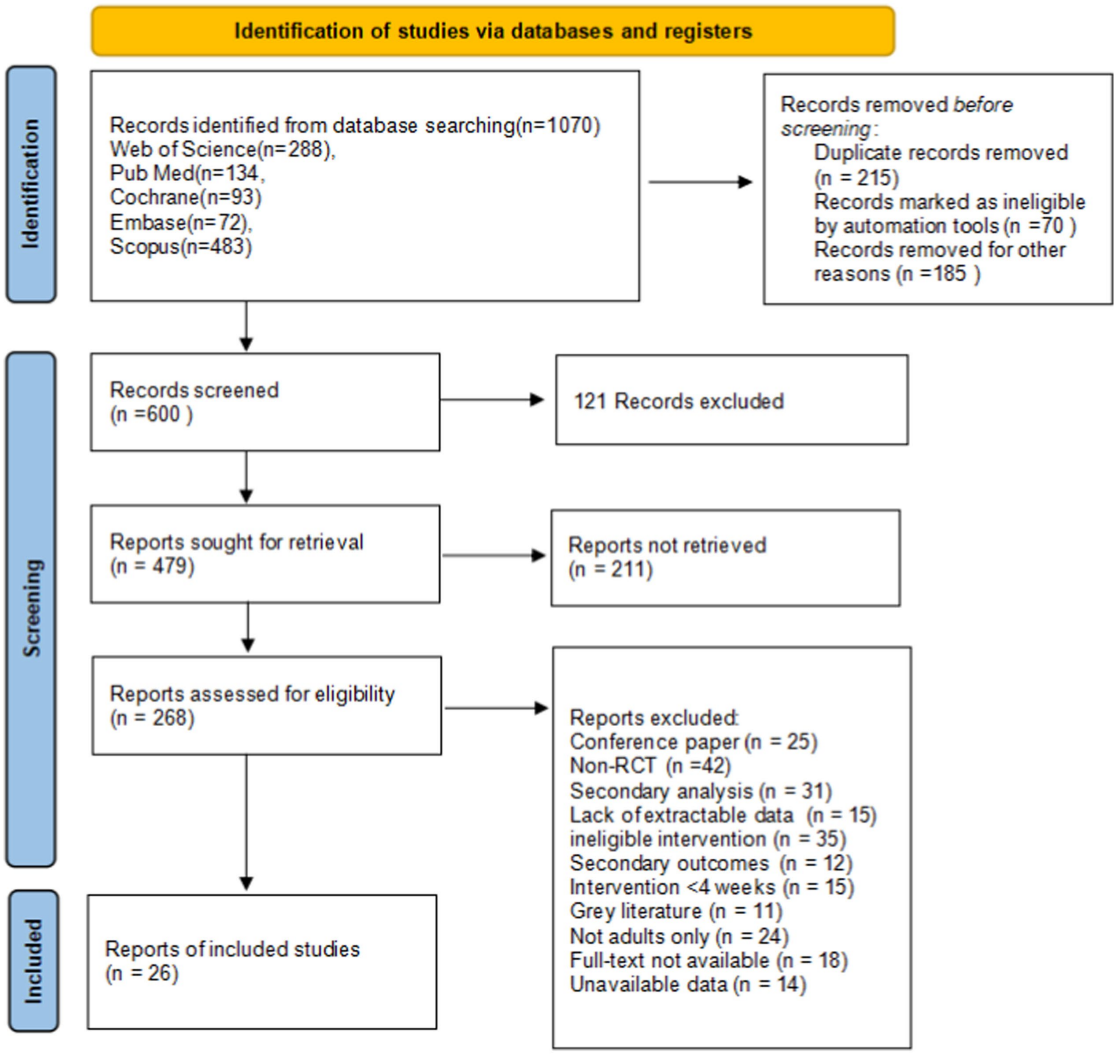
PRISMA flow diagram of the study process.

A total of 1,070 articles were identified from various databases and additional sources. After duplicates were removed, 215 articles were excluded, leaving 855 articles for further screening. Ultimately, 26 studies were included in the analysis, with a total of 1,507 participants. The screening process is shown in Figure 1.

### 3.2 Basic characteristics of included studies

Among the 26 studies included, the exercise interventions in the experimental groups primarily involved core stability training, combined training, Pilates, yoga, qigong, suspension training, Swiss ball exercises, strength training, perturbation therapy, sit-up exercises, and tai chi. The intervention durations varied, ranging from 4, 6, 8, 12, 13, and ≥16 weeks. The frequency of interventions ranged from 1–2 sessions, 3 sessions, 5 sessions, to 7 sessions per week. Intervention durations ranged from 15–30 min, 40 min, 45 min, 50 min, to ≥60 min. The detailed characteristics of the included studies are presented in Table 2.

**Table 2:** Basic characteristics of included literature.

Author & year	Country	*N*	Mean age (years)	Instrument	Dose
Akhtar et al. (14)	Pakistan	C = 55	45.50 ± 6.61	Usual care	60 min, 2times, 6 weeks
		E = 53	46.39 ± 7.43	Core stabilization exercise	
Arampatzis et al. (56)	Germany	C = 20	31.4 ± 5.5	Usual care	90 min, 2times, 13 weeks
		E = 20	31.9 ± 6.0	Disturbance exercise	
Gladwell et al. (17)	UK	C = 14	45.9 ± 8.0	Usual care	60 min, 1time, 6 weeks
		E = 20	36.9 ± 8.1	Pilates	
Bae et al. (79)	Korea	C = 18	32.4 ± 10.7	Abdominal crunch exercise	30 min, 3times, 12 weeks
		E = 18	32.7 ± 6.1	Core stabilization exercise	
Oh et al. (80)	Korea	C = 10	44.2 ± 2.70	Usual care	30 min, 5times, 12 weeks
		E1 = 10	46.0 ± 3.37	Swiss ball	
		E2 = 10	46.2 ± 3.22	Sling exercise	
Cho et al. (81)	Korea	C = 15	44.0 ± 6.7	Usual care	40 min, 3times, 6 weeks
		E = 15	48.1 ± 6.9	Core stabilization exercise	
Cho et al. (82)	Korea	C = 15	36.5 ± 7.7	Usual care	40 min, 3times, 4 weeks
		E = 15	38.1 ± 7.9	Core stabilization exercise	
Hwangbo et al. (83)	Korea	C = 15	34.0 ± 2.9	Usual care	50 min, 3times, 6 weeks
		E = 15	34.5 ± 4.0	Core stabilization exercise	
Liu et al. (41)	Chinese	C = 13	60.67 ± 2.58	Usual care	60 min, 3times, 12 weeks
		E1 = 15	58.13 ± 5.38	Tai Chi	
		E2 = 15	58.4 ± 5.08	Core stabilization exercise	
Williams et al. (84)	Greece	C = 26	35.2 ± 9.7	Usual care	50 min, 2times, 8 weeks
		E = 29	39.2. ± 11.4	Core stabilization exercise	
Kumar (12)	India	C = 9	22.5 ± 1.09	Usual care	15 min, 8 weeks
		E = 9		Core stabilization exercise	
Lee and Kang (85)	Korea	C = 6	43.3 ± 9.9	Usual care	50 min, 2times, 12 weeks
		E1 = 15	42.7 ± 13.4	Strength exercise	
		E2 = 15	46.7 ± 8.1	Combination exercise	
Michaelson et al. (86)	Sweden	C = 35	52.1 ± 17.16	Usual care	120 min, 1time, 8 weeks
		E = 35	49.3 ± 14.0	Core stabilization exercise	
Noormohammadpour et al. (87)	Iran	C = 10	41.3 ± 6.4	Usual care	8 weeks
		E = 10	43.3 ± 7.5	Core stabilization exercise	
Roh et al. (88)	Korea	C = 49	50.5 ± 9.1	Usual care	30 min, 3times, 12 weeks
		E = 53	49.5 ± 10.6	Sling exercise	
Shamsi et al. (15)	Iran	C = 20	38.5 ± 11.9	Usual care	40 min, 3times, 6 weeks
		E = 19	47.7 ± 10.4	Core stabilization exercise	
Tang et al. (18)	Chinese	C = 41	43.6 ± 6.4	Usual care	30 min, 7times, 6 weeks
		E = 41	41.7 ± 5.6	Core stabilization exercise	
Tekur et al. (89)	India	C = 40	48.0 ± 4.0	Usual care	45 min, 7times, 4 weeks
		E = 40	49.0 ± 3.6	Core stabilization exercise	
Teut et al. (30)	Germany	C = 57	72.6 ± 6.0	Usual care	45 min, 2times, 12 weeks
		E1 = 61	73.0 ± 5.6	Yoga	
		E2 = 58	72.4 ± 5.7	Qigong	
Ulger et al. (67)	Turkey	C = 12	55.08 ± 2.67	Core stabilization exercise	60 min, 2times, 8 weeks
		E = 16	47.12 ± 7.07	Yoga	
Ulger et al. (90)	Turkey	C = 56	41.6 ± 12.9	Usual care	60 min, 3times, 6 weeks
		E = 57	48.4 ± 1.86	Core stabilization exercise	
Williams et al. (91)	USA	C = 47	47.6 ± 1.47	Usual care	30 min, 7times, 24 weeks
		E = 43	48.4 ± 1.86	Yoga	
Yoo and Lee (57)	Korea	C = 15	20.5 ± 0.5	Usual care	45 min, 3times, 4 weeks
		E = 15	20.1 ± 0.7	Sling exercise	
Zhang et al. (92)	Chinese	C = 46	51.62 ± 4.03	Usual care	40 min, 7times, 8 weeks
		E = 46	48.71 ± 3.8	Core stabilization exercise	
Koumantakis et al. (13)	Greece	C = 26	35.2 ± 9.7	Usual care	40–60 min, 2times, 8 weeks
		E = 29	39.2 ± 11.4	Core stabilization exercise	
You et al. (16)	Korea	C = 20	51.30 ± 7.01	Usual care	40 min, 3times, 8 weeks
		E = 20	50.35 ± 9.26	Core stabilization exercise	

E, Experimental group; C, Control group.

### 3.3 Quality assessment of included studies

**Figure 2:**
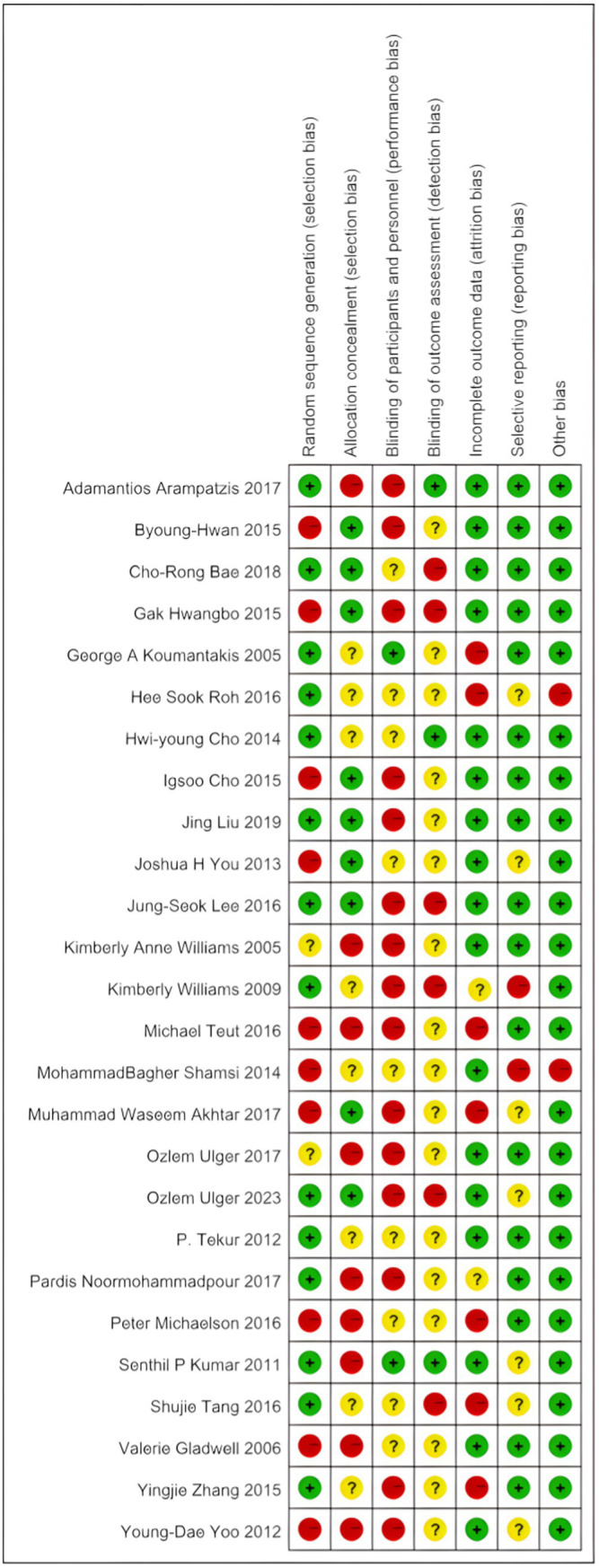
Bias risk diagram for each item.

**Figure 3:**
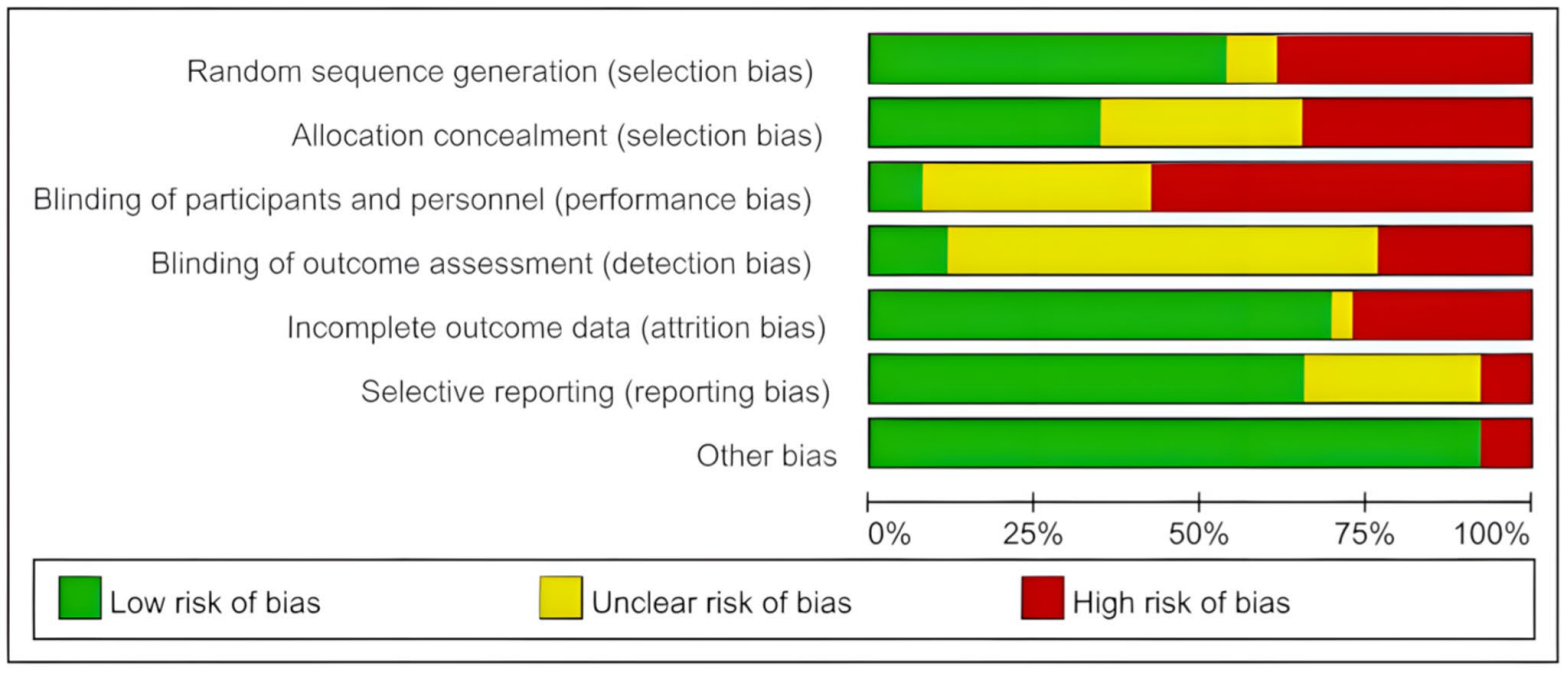
Overall bias risk diagram.

The quality of the included studies was comprehensively evaluated using RevMan 5.4 software and the Cochrane risk of bias assessment tool (4, 31). The Cochrane risk of bias results for each study are shown in Figure 2, while Figure 3 presents a visual representation of the overall distribution of bias risk. Studies that employed randomization for allocation were classified as having a low risk of selection bias. In contrast, studies that either did not employ randomization or did not report the randomization process were classified as having a high risk of bias. Due to the inclusion of multiple exercise interventions and the inability of participants to remain blinded to treatment allocation, the majority of studies were classified as having a high risk of bias for patient blinding. The detailed risk of bias assessments for each study are provided in Table 3.

**Table 3:** Risk of bias assessment of included studies (*n* = 26) examining the efficacy of exercise training in patients with nonspecific chronic low back pain.

Study	Random sequence generation (selection bias)	Allocation concealment (selection bias)	Blinding of patients and personnel (performance bias)	Blinding of outcome assessment (detection bias)	Incomplete outcome data (attrition bias)	Selective outcome reporting (reporting bias)	Any other bias
Akhtar et al. (14)	High	Low	High	Unclear	High	Unclear	Low
Arampatzis et al. (56)	Low	High	High	Low	Low	Low	Low
Gladwell et al. (17)	High	High	Unclear	Unclear	Low	Low	Low
Bae et al. (79)	Low	High	Unclear	High	Low	Low	Low
Oh et al. (80)	High	Low	High	High	Low	Low	Low
Cho et al. (81)	High	Low	High	Unclear	Low	Low	Low
Cho et al. (82)	Low	Unclear	Unclear	Unclear	Low	High	High
Hwangbo et al. (83)	High	High	High	Low	Low	Low	Low
Liu et al. (41)	Low	Low	High	Unclear	Low	Low	Low
Williams et al. (84)	Unclear	High	High	Unclear	Low	Low	Low
Kumar (12)	Low	High	Low	Low	Low	Unclear	Low
Lee and Kang (85)	Low	Low	High	High	Low	Low	Low
Michaelson et al. (86)	High	High	Unclear	Unclear	High	Low	Low
Noormohammadpour et al. (87)	Low	High	High	Unclear	Low	Low	Low
Roh et al. (88)	Low	Unclear	Unclear	Unclear	High	Unclear	High
Shamsi et al. (15)	High	High	High	Low	Low	Low	Low
Tang et al. (18)	Low	Unclear	Unclear	High	High	Unclear	Low
Tekur et al. (89)	Low	Unclear	Unclear	Unclear	Low	Low	Low
Teut et al. (30)	High	High	High	Unclear	High	Low	Low
Ulger et al. (67)	Low	Low	High	High	Low	Unclear	Low
Ulger et al. (90)	Unclear	High	High	Unclear	Low	Low	Low
Williams et al. (91)	Low	Unclear	High	High	Unclear	High	Low
Yoo and Lee (57)	High	High	High	Unclear	Low	Unclear	Low
Zhang et al. (92)	Low	Unclear	High	Unclear	High	Low	Low
Koumantakis et al. (13)	Low	Unclear	Low	Unclear	High	Low	High
You et al. (16)	High	Low	Unclear	Unclear	Low	Unclear	Low

### 3.4 Network meta-analysis results

#### 3.4.1 Network plot

**Figure 4:**
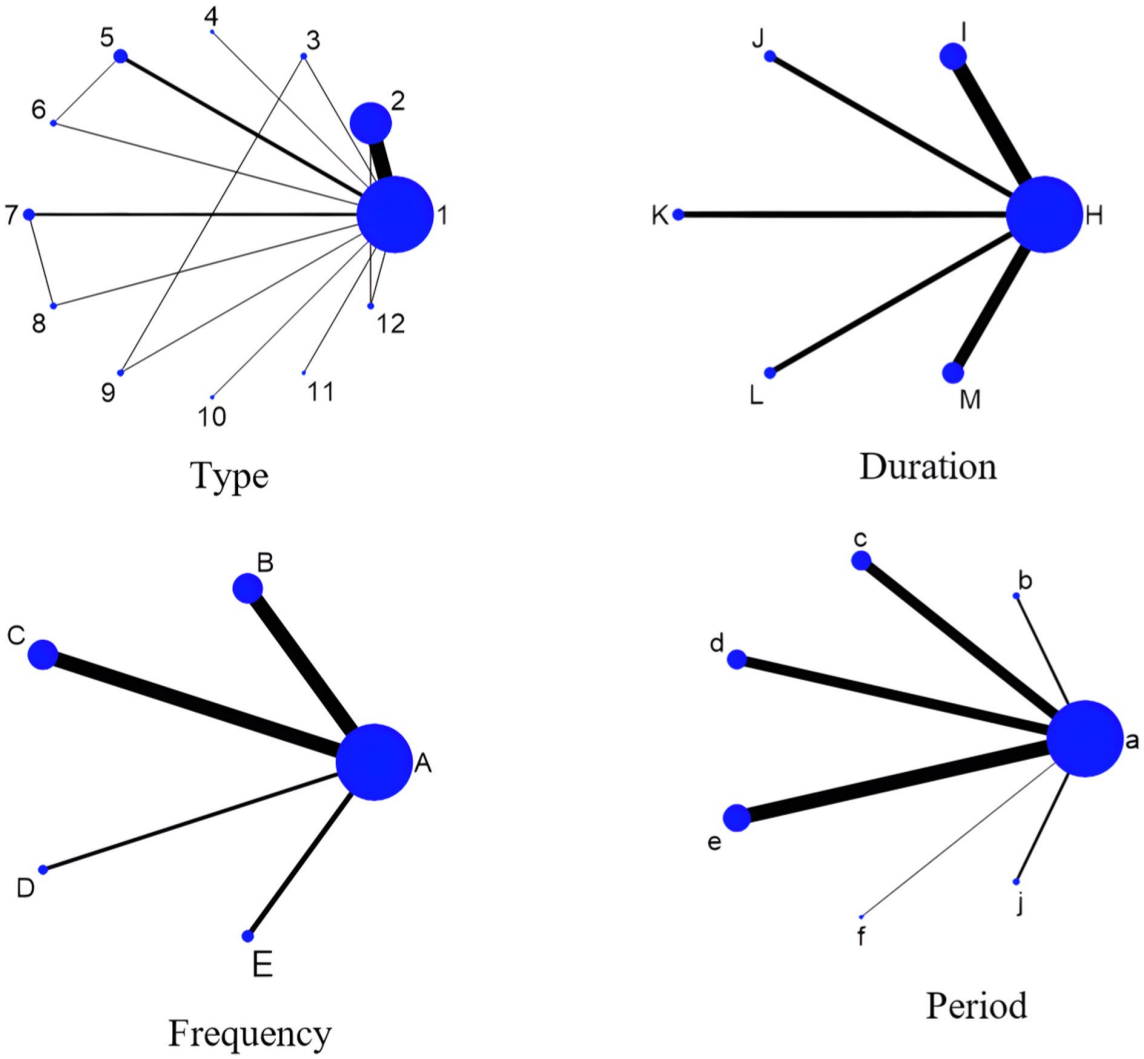
Network plot of the impact of exercise prescription elements on chronic low back pain control in adults. 1, con; 2, Core stabilization exercise; 3, Combination exercise; 4, Pilates; 5, Yoga; 6, Qigong; 7, Sling exercise; 8, Swiss ball; 9, Strength exercise; 10, Disturbance exercise; 11, Abdominal crunch exercise A; 12, Taichi; A, con; B, 1–2 times; C, 3 times; D, 5 times; E, 7 times; H, con; I, 15–30 min; J, 40 min; K, 45 min; L,50 min; M, ≥60 min; a, con; b, 4 weeks; c, 6 weeks; d, 8 weeks; e, 12 weeks; f, 13 weeks; j, ≥16weeks.

A network plot is used to illustrate the direct and indirect comparative relationships among multiple interventions. In this plot, the connecting lines represent the direct comparisons between two interventions based on the original studies. The size of each node (i.e., point) corresponds to the sample size of the respective intervention in the study; larger sample sizes are represented by larger nodes. The thickness of the connecting lines reflects the amount of evidence for the direct comparison between the two interventions; thicker lines indicate a greater number of studies making direct comparisons between the two interventions. The specific network plot is presented in Figure 4. (Note: When an element refers to a range, the lower limit of the range is used).

#### 3.4.2 Results of pairwise comparisons of elements of exercise prescription

**Figure 5:**
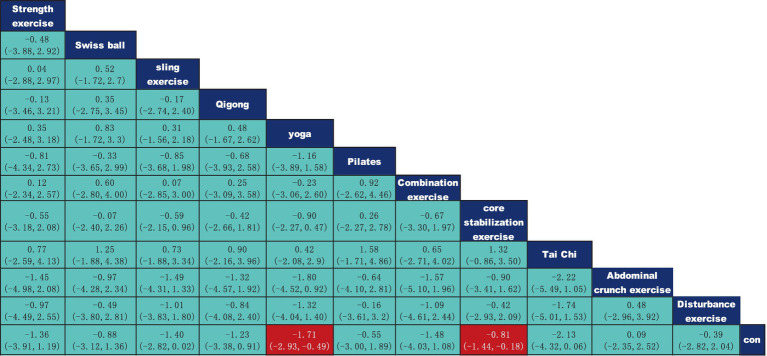
League table of pairwise comparisons of intervention effects for different elements of exercise types.

**Figure 6:**
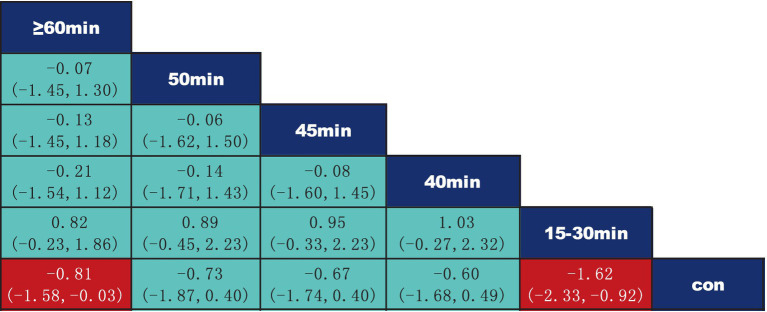
League table of pairwise comparisons of intervention effects for different elements of exercise duration.

**Figure 7:**
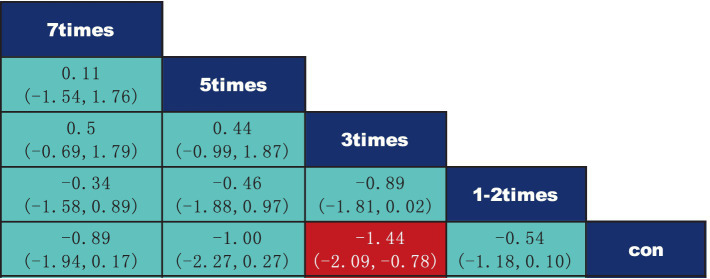
League table of pairwise comparisons of intervention effects for different elements of exercise frequency.

**Figure 8:**
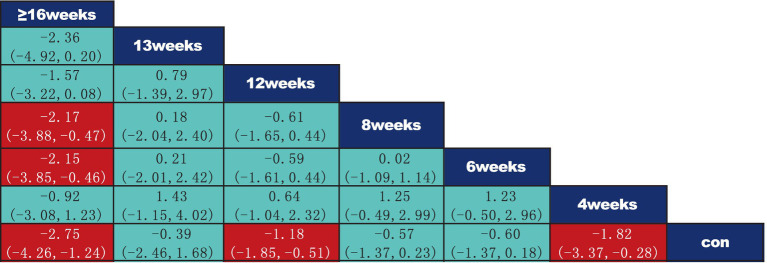
League table of pairwise comparisons of intervention effects for different elements of exercise period. The red numbers are statistically significant.

According to the data presented in Figure 5, both Yoga (SMD = −1.71, 95% CI: −2.93 to −0.49, *p* < 0.05) and Core Stability Training (SMD = −0.81, 95% CI: −1.44 to −0.18, *p* < 0.05) demonstrated significant improvement compared to the control group, showing substantial effects on pain intensity reduction. Data from Figure 6 indicate that intervention durations of 15–30 min (SMD = −1.62, 95% CI: −2.33 to −0.92) and ≥60 min (SMD = −0.81, 95% CI: −1.58 to −0.03) also significantly outperformed the control group. As shown in Figure 7, an intervention frequency of 3 times per week (SMD = −1.44, 95% CI: −2.09 to −0.78) demonstrated significantly better outcomes than the control group. Figure 8 presents data indicating that intervention periods of 4 weeks (SMD = −1.82, 95% CI: −3.37 to −0.28), 12 weeks (SMD = −1.18, 95% CI: −1.85 to −0.51), and ≥16 weeks (SMD = −2.75, 95% CI: −4.26 to −1.24) demonstrated significantly superior effects compared to the control group. Furthermore, interventions lasting ≥16 weeks also significantly outperformed those of 12 weeks (SMD = −2.17, 95% CI: −3.58 to −0.47) and 6 weeks (SMD = −2.18, 95% CI: −3.85 to −0.45). No significant differences were found in other pairwise comparisons.

#### 3.4.3 Probability ranking of the most effective interventions for elements of exercise prescription

**Figure 9:**
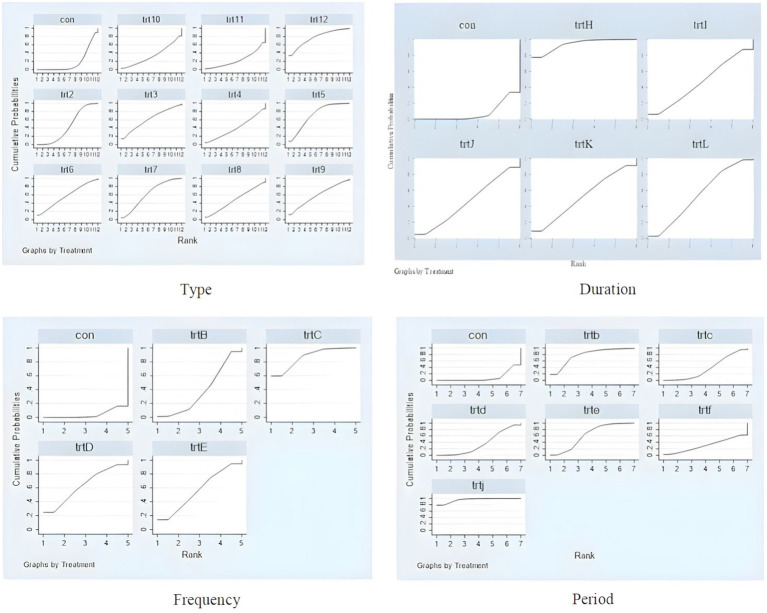
Probability ranking chart of intervention effects for different elements of exercise prescription dose. Type, con; trt2, Core stabilization exercise; trt3, Combination exercise; trt4, Pilates; trt5, Yoga; trt6, Qigong; trt7, Sling exercise; trt8, Swiss ball; trt9, Strength exercise; trt10, Disturbance exercise; trt11, Abdominal crunch exercise; trt12, Taichi; Duration, con; trtH, 15–30 min; trtI, 40 min; trtJ, 45 min; trtK, 45 min; trtL, ≥60 min; Frequency, con; trtB, 1–2 times; trtC, 3 times; trtD, 5 times; trtE, 7 times; Period, con; trtb, 4 weeks; trtc, 6 weeks; trtd, 8 weeks; trte, 12 weeks; trtf, 13 weeks; trtj, ≥16 weeks.

Based on the SUCRA values presented in Table 4 and Figure 9, the ranking of different exercise intervention types according to their effectiveness in improving chronic low back pain in adults is as follows: Tai Chi (SUCRA = 77.4) > Yoga (SUCRA = 72.1) > Sling Exercise (SUCRA = 63) > Combination Exercise (SUCRA = 61.6) > Strength Exercise (SUCRA = 59.2) > Qigong (SUCRA = 57.5) > Swiss Ball (SUCRA = 48) > Core Stabilization Exercise (SUCRA = 44.8) > Pilates (SUCRA = 40.5) > Disturbance Exercise (SUCRA = 34.5) > Abdominal Crunch Exercise (SUCRA = 23.4). In terms of intervention duration, the ranking of effectiveness for improving chronic low back pain in adults is as follows: 15–30 min (SUCRA = 94.6) > ≥60 min (SUCRA = 55.9) > 50 min (SUCRA = 51.9) > 45 min (SUCRA = 47.7) > 40 min (SUCRA = 42.5). The ranking of the effects of different intervention frequencies on the improvement of chronic low back pain in adults is as follows: 3 times per week (SUCRA = 87) > 5 times per week (SUCRA = 63.6) > 7 times per week (SUCRA = 56.7) > 1–2 times per week (SUCRA = 38.4). The ranking of intervention duration effectiveness is as follows: ≥16 weeks (SUCRA = 95.4) > 4 weeks (SUCRA = 78.1) > 12 weeks (SUCRA = 62.7) > 6 weeks (SUCRA = 37.4) > 8 weeks (SUCRA = 36.2) > 13 weeks (SUCRA = 31.1).

**Table 4:** SUCRA values for the effectiveness of interventions by exercise prescription elements.

Rank	Type	SUCRA	Frequency	SUCRA	Duration	SUCRA	Period	SUCRA
1	Taichi	77.4	≥16 weeks	95.4	15–30 min	94.6	3times	87.0
2	Yoga	72.1	4 weeks	78.1	≥60 min	55.9	5times	63.6
3	Sling exercise	63.0	12 weeks	62.7	50 min	51.9	7times	56.7
4	Combination exercise	61.6	6 weeks	37.4	45 min	47.7	1-2times	38.4
5	Strength exercise	59.2	8 weeks	36.2	40 min	42.5	con	4.4
6	Qigong	57.5	13 weeks	31.1	con	7.3		
7	Swiss ball	48.0	Con	9.1				
8	Core stabilization exercise	44.8						
9	Pilates	40.5						
10	Disturbance exercise	34.5						
11	Abdominal crunch exercise	23.4						
12	Con	18.0						

#### 3.4.4 Sensitivity analysis

A leave-one-out sensitivity analysis was performed to assess the robustness of the results. The analysis demonstrated that, after removing any individual study, the pooled effect size fluctuated within the range of −1.059 to −0.845. This indicates that the exclusion of any individual study had a minimal impact on the overall pooled effect, confirming the stability and robustness of the findings in this analysis (Table 5).

#### 3.4.5 Publication bias assessment

**Figure 10:**
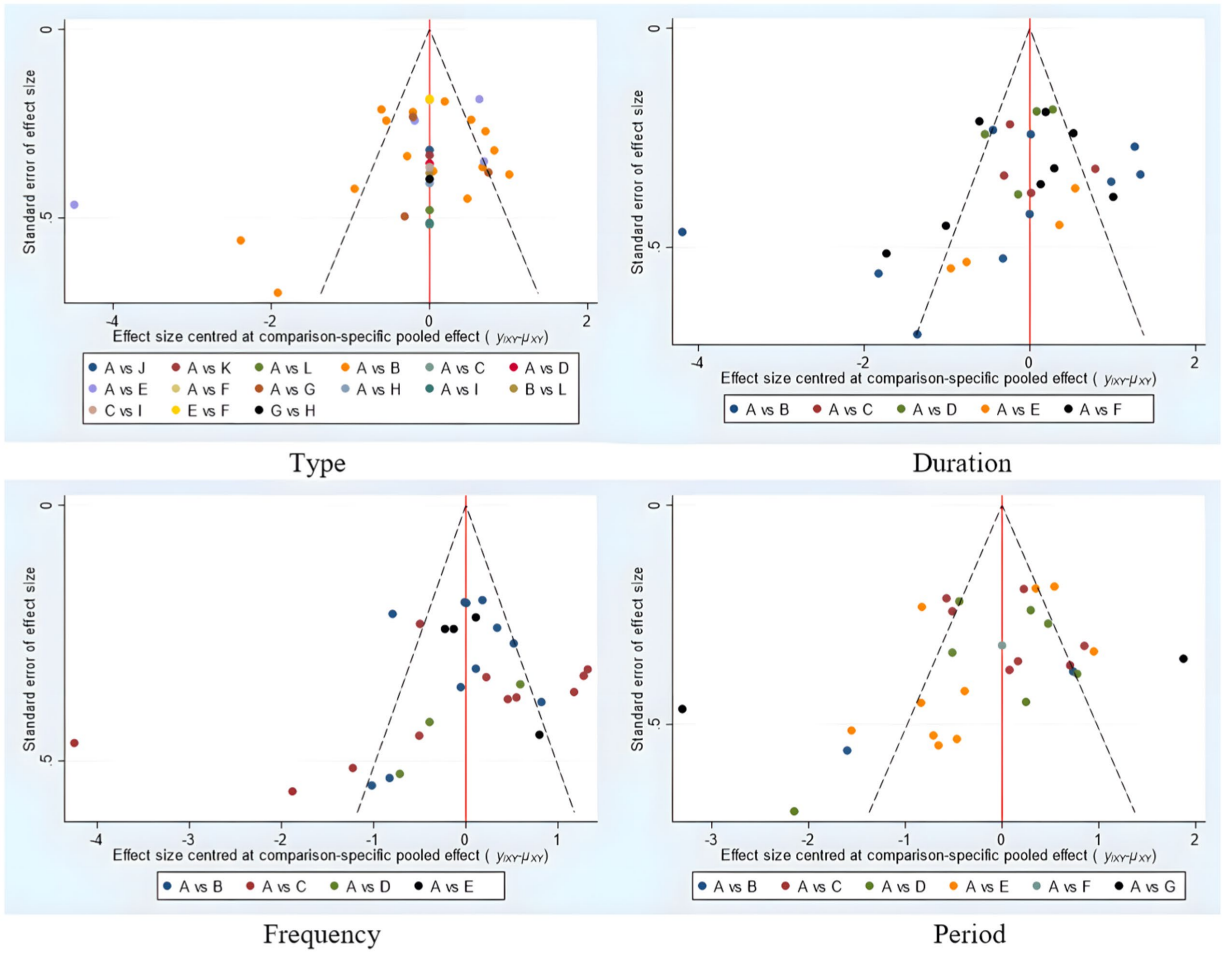
Funnel plot of intervention effects for different elements of exercise prescription dose. Type: A, con; B, Core stabilization exercise; C, Combination exercise; D, Pilates; E, Yoga; F, Qigong; G, Sling exercise; H, Swiss ball; I, Strength exercise; J, Disturbance exercise; K, Abdominal crunch exercise; L, Taichi; Duration: A, con; B, 15–30 min; C, 40 min; D, 45 min; E, 50 min, E, ≥60 min; Frequency: A, con; B, 1–2 times; C, 3 times; D, 5 times; E, 7 times; Period: A, con; B, 4 weeks; C, 6 weeks; D, 8 weeks; E, 12 weeks; F, 13 weeks; G ≥16 weeks.

As shown in Figure 10, the funnel plots for all indicators were nearly symmetrical, with the majority of points located in the upper part of the funnel and only a few points falling outside the funnel. This suggests that the likelihood of publication bias in this study is low. Furthermore, Egger’s test for publication bias yielded a *p*-value of 0.161, indicating no significant evidence of publication bias. However, caution is advised when interpreting these results, and further studies may be needed to confirm the findings.

## 4 Discussion

Despite the existence of several recent systematic reviews and meta-analyses discussing the effects of exercise interventions on chronic low back pain (CLBP) in adults, our network meta-analysis is the first to investigate the effects of exercise prescription components on CLBP outcomes (32–35). Additionally, we ranked the impact of different exercise types, frequencies, durations, and periods on adult chronic low back pain, which will aid in identifying the most effective exercise interventions for this population. This information is crucial for formulating the most appropriate and beneficial intervention strategies.

Our network meta-analysis identified that the most effective exercise for alleviating chronic low back pain in adults may be Tai Chi. The optimal intervention duration is likely 15–30 min, the best frequency is 3 times per week, and the most beneficial intervention period is likely ≥16 weeks.

Firstly, our findings suggest that Tai Chi is an effective exercise intervention that can significantly improve symptoms of CLBP in adults (36, 37). However, this contradicts existing research findings. Existing meta-analysis results suggest that Pilates, aerobic exercise, and resistance training may be the best interventions for adult chronic low back pain (38–40). The superiority of Tai Chi in our analysis may be attributed to its unique exercise approach. Tai Chi is a slow, continuous, and mindful form of exercise that helps regulate muscle tension, effectively alleviating the tension-type pain associated with chronic low back pain. Furthermore, Tai Chi emphasizes core stability, body posture, muscle coordination, strength, joint flexibility, and breathing regulation. By training these physical components in an integrated manner, Tai Chi enhances overall body function and comfort, contributing to the relief of chronic low back pain (37). The smooth, flowing, and coordinated movements of Tai Chi, coupled with its emphasis on controlled breathing and mental focus, are thought to improve core stability and lumbar proprioception (41–43). Furthermore, the circular and spiral movements in Tai Chi are thought to gently stretch and strengthen the lumbar muscles and connective tissues, addressing muscle imbalances and stiffness associated with CLBP (44).

Tai Chi, as a traditional mind–body exercise, demonstrated a high efficacy score in this study. During the bias risk assessment for Tai Chi, we found that the randomization method was well-implemented, with both random sequence generation and allocation concealment assessed as low risk. This indicates that the study adhered to rigorous standards for randomization and allocation control. However, there was a higher risk associated with the blinding of trial participants and operators, which may have influenced the evaluation of the intervention’s effectiveness. Additionally, the blinding status of the assessors is unclear, which may introduce subjectivity into the evaluation results. In terms of data completeness and accuracy, there was minimal missing data, leading to a low-risk assessment for these factors. Furthermore, there was no selective reporting of outcomes in the study, contributing to the overall low risk of bias. Other potential sources of bias were also evaluated as low-risk. Overall, the study’s risk of bias was relatively low. While the insufficient blinding of participants and operators may have influenced the evaluation of treatment effects, sensitivity analyses excluding high-risk bias studies indicated that the efficacy of Tai Chi remained stable, further confirming its effectiveness.

Nevertheless, caution should be exercised when promoting Tai Chi as a therapeutic intervention, particularly considering that other forms of exercise, such as Pilates, core stability training, and yoga, also demonstrate significant therapeutic effects and have been recommended in several clinical guidelines (45). Each type of exercise has advantages and is worth trying in therapy. However, our study found that although the effect of tai chi seems to be good, it may be due to factors such as cultural background and exercise habits, which affect the results of the overall analysis. Therefore, when choosing exercises for patients with chronic low back pain, we should not only look at the ranking of exercise effectiveness, but also consider the patients themselves, such as whether they can do it, whether they like it, and whether they can stick to it. Only by combining these factors with the exercise rankings can we find exercises that are both effective and easy to adhere to, and ultimately achieve the best treatment results.

Secondly, the results of this study suggest that the optimal intervention duration for improving chronic low back pain in adults is 15–30 min, consistent with previous studies (46). This duration ensures sufficient stimulation to promote muscle relaxation, enhance core muscle strength, and improve joint flexibility, while avoiding muscle fatigue or discomfort that may arise from prolonged duration, thus effectively alleviating back pain symptoms and promoting recovery (47, 48). This phenomenon can be reasonably explained from multiple theoretical perspectives. According to the “Supercompensation Theory,” the body undergoes a recovery phase after moderate stimulation, eventually surpassing its previous level (49). In this study, despite the relatively short total intervention duration, this precise stimulation enabled the body to quickly adapt and recover, avoiding fatigue and injury associated with prolonged exercise. Through deep breathing and gentle movements, participants could further relax their bodies and consolidate the benefits of the practice (50–52). Furthermore, the ‘Adaptation Efficiency Hypothesis’ offers another perspective. This hypothesis posits that, within a given time frame, the body adapts most effectively to stimuli that best promote its overall function (53). In this study, the 15-30-min intervention duration may represent the optimal time window for triggering the body’s adaptive mechanism and promoting recovery (54, 55).

The study also found that a three-times-per-week intervention schedule significantly improved chronic low back pain (CLBP) in adults, consistent with existing studies (56–58). Further analysis reveals why a three-times-per-week intervention schedule has been shown to be more effective from several perspectives. From a physiological adaptation standpoint, the body requires time to recover after exercise stimulation to reach a supercompensation state before the next session (59–61). Excessively frequent interventions may lead to overuse of muscles and joints, increasing the risk of injury and potentially inhibiting the body’s natural recovery process (62). In contrast, a three-times-per-week schedule provides adequate stimulation to promote recovery while offering necessary recovery time, allowing the body to reach its optimal state before the next intervention (61). From a behavioral compliance perspective, a three-times-per-week schedule may be more acceptable and easier for patients to adhere to (63). An overly frequent training program may bring more stress to patients’ lives, thus reducing their motivation to participate in training and their willingness to adhere to it in the long term (64). On the contrary, moderate training frequency not only reduces patients’ psychological burden, but also enhances their confidence in treatment (65). In addition, a study of patients with chronic low back pain showed that patients who performed core stability training 3 times per week performed well in terms of pain relief and functional improvement, with no significant difference in outcomes compared with patients who trained 7 times per week (66). This result emphasizes the importance of moderate interventions and suggests that we do not have to overdo the high frequency of training to achieve good therapeutic results when developing a training program.

Finally, the results of this study indicate that an intervention period exceeding 16 weeks is optimal for improving chronic low back pain in adults (67). This contrasts with previous findings, which suggest that an intervention period of ≤8 weeks is the most effective for treating chronic low back pain in adults (68). However, chronic low back pain is often accompanied by long-term pathological changes, such as muscle imbalances, ligament laxity, and intervertebral disc degeneration, which cannot be fully reversed within a short time frame (69). Therefore, a longer intervention period provides the body with sufficient time to adapt and produce sustained therapeutic effects (49). As the intervention period lengthens, the body gradually attains a new state of balance, leading to more stable pain relief and functional recovery (53). Long-term interventions not only address physical symptoms but also promote the development of healthy lifestyle and exercise habits (70). Through consistent Tai Chi practice, patients can gradually integrate exercise into their daily routines, establishing positive health behavior patterns. This behavioral change not only alleviates chronic low back pain symptoms but also plays a crucial role in preventing relapse (71). During long-term intervention, patients benefit not only from physical improvements but also from psychological support provided by coaches and peers (72). This behavior is very helpful in improving patients’ negative emotions and can significantly improve their quality of life (73). Therefore, this study concludes that intervention cycles longer than 16 weeks are more effective than short-term interventions. This is because a longer intervention period can provide enough time for full physical recovery.

## 5 Limitations

Firstly, because most of the included studies did not adequately implement blinding, bias may have been introduced, potentially compromising the objectivity and reliability of the findings. Secondly, due to resource and time constraints, this study did not incorporate registered but unpublished clinical trial data, which may have resulted in incomplete data. Moreover, the studies included were predominantly focused on the Asian region, which may limit the generalizability of the findings and their applicability to broader populations. Despite these limitations, this study provides valuable insights, and future research should aim to include data from a greater number of randomized controlled trials to improve the comprehensiveness and reliability of the conclusions.

## 6 Implications for research

In the 2017 guidelines, the American College of Physicians (ACP) recommended exercise as the first-line non-pharmacological treatment for chronic non-specific or radicular low back pain. Specifically, the ACP endorsed Tai Chi, yoga, spinal manipulation, massage, and acupuncture as effective therapeutic methods (74). The National Institute for Health and Care Excellence (NICE), in its 2010 guidelines, also recommended moderate exercise as the first choice for treating chronic low back pain. The guidelines emphasized that patients should be encouraged to remain active, avoid prolonged bed rest, and engage in moderate exercises, such as walking, swimming, or yoga (75). Although the specific recommendations of these two guidelines vary, our findings largely align with the recommended interventions. Notably, the NICE guidelines stress the importance of considering the patient’s individual needs, preferences, and abilities when selecting the type of exercise, and our results align with the low-intensity exercise interventions (such as Tai Chi and yoga) recommended by both the ACP and NICE. We found that Tai Chi, as a low-risk, low-intensity exercise, is highly effective in alleviating chronic low back pain, supporting the recommendations of these clinical guidelines (76).

However, despite the consistency of our study with the ACP and NICE guidelines, there are notable differences in some aspects. For instance, the NICE guidelines highlight the importance of multidisciplinary interventions and individualized treatment plans, while our study focuses primarily on Tai Chi as a sole intervention. Another difference is that the ACP guidelines emphasize short-term effects, while our study provides an evaluation of long-term interventions lasting more than 16 weeks, emphasizing the importance of sustained exercise. Furthermore, the World Health Organization (WHO) guidelines published in 2020 also explicitly recommend exercise interventions as the first-line treatment for chronic low back pain, advocating for aerobic and stretching exercises aimed at strengthening muscles, improving function, and reducing pain (77). Additionally, the Australian Commission on Safety and Quality in Health Care (ACSQHC) emphasized in its 2017 guidelines that exercise interventions are crucial for treating low back pain, recommending moderate exercise to alleviate pain symptoms (78).

## 7 Implications for clinical practice

Our study suggests that exercise interventions, particularly Tai Chi, may be among the most effective methods for alleviating pain in patients with chronic low back pain (LBP). Tai Chi, as a low-intensity, low-risk exercise, not only helps strengthen and improve the flexibility of the lumbar muscles but also effectively reduces pain through mind–body relaxation techniques. Long-term commitment to exercise is crucial, with an optimal intervention period potentially exceeding 16 weeks to achieve more significant therapeutic effects. Although we provide exercise prescription recommendations for public health, many chronic LBP patients still require personalized exercise plans tailored to individual differences. Therefore, we recommend improving the pre-exercise health assessment system, which should comprehensively evaluate a patient’s physical abilities, potential risks, health status, exercise goals, and preferences. Based on these assessments, personalized exercise plans should be developed. Furthermore, regular evaluation of exercise outcomes, along with adjustments based on the latest research evidence, will ensure the ongoing effectiveness of the exercise prescription. Additionally, regular evaluations of exercise effectiveness should be conducted, with adjustments made based on the latest research evidence to ensure the ongoing effectiveness of the exercise prescription and further reduce patients’ reliance on medication and surgical treatments.

## 8 Conclusion

This study included 26 studies and performed a network meta-analysis to compare the effects of different exercise prescriptions on improving chronic low back pain (CLBP) in adults. Limited evidence suggests that an exercise prescription with a duration of 15–30 min, a frequency of three times per week, a duration exceeding 16 weeks, and Tai Chi as the intervention type may be the most effective for improving chronic low back pain in adults. This finding is an important guide to help adults choose the most appropriate exercise intervention program. However, due to the small number of studies included in this study and the fact that most of the studies focused on the Asian region, we still need to remain cautious in interpreting these results. Future studies need to increase the number of studies and work to address current limitations to improve the comprehensiveness and reliability of the evidence.
