# Supplementary material for: Exercise prescription for improving chronic low back pain in adults: a network meta-analysis
Source: Front Public Health. 2025 May 30;13:1512450. doi: 10.3389/fpubh.2025.1512450 (PMC12162995; doi:10.3389/fpubh.2025.1512450)
Supplement: Supplementary file 1 [file Data_Sheet_1.docx]

Supplementary Material

# Supplementary Data

Supplementary Material should be uploaded separately on submission. Please include any supplementary data, figures and/or tables.

Supplementary material is not typeset so please ensure that all information is clearly presented, the appropriate caption is included in the file and not in the manuscript, and that the style conforms to the rest of the artic

# 3 Supplementary Figures and Tables

## 3.1 Supplementary Figures


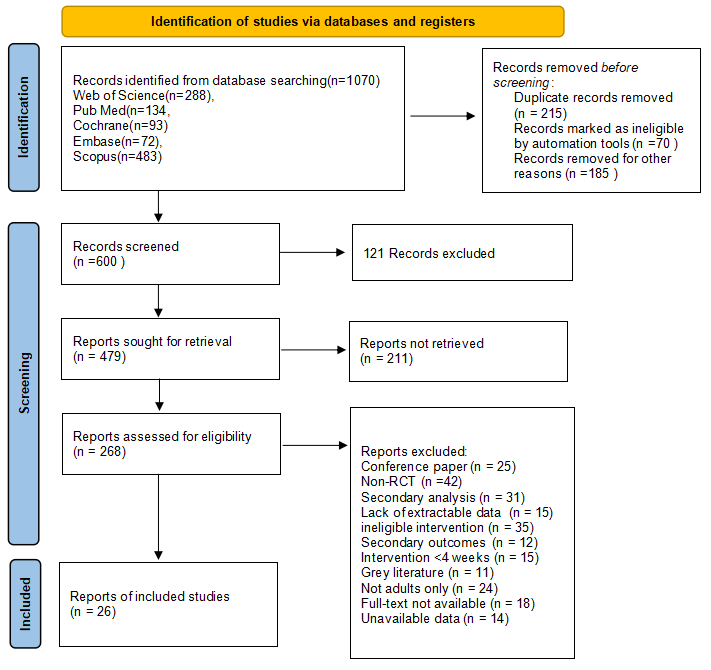


Figure 1 PRISMA flow diagram of the study process

## 3.3 Supplementary Figures


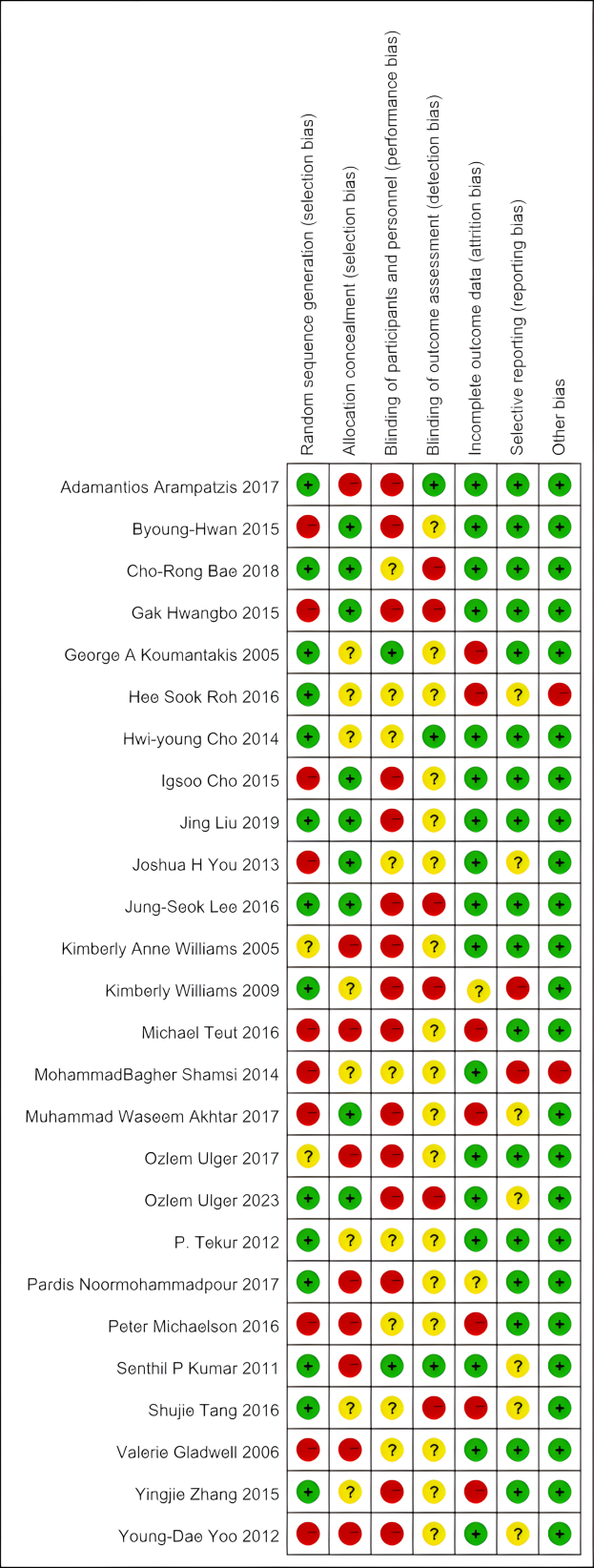


Figure2 Bias Risk Diagram for Each Item


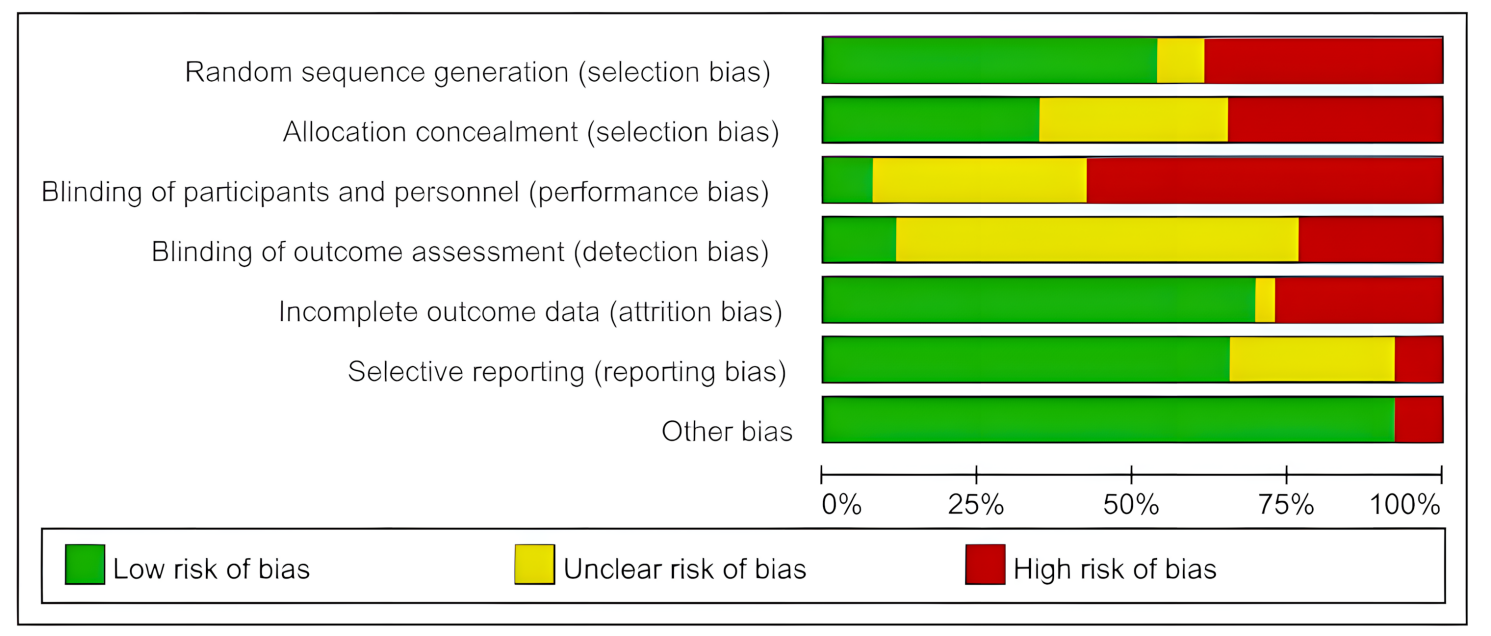


Figure3 Overall Bias Risk Diagram

## 3.4.1 Supplementary Figures


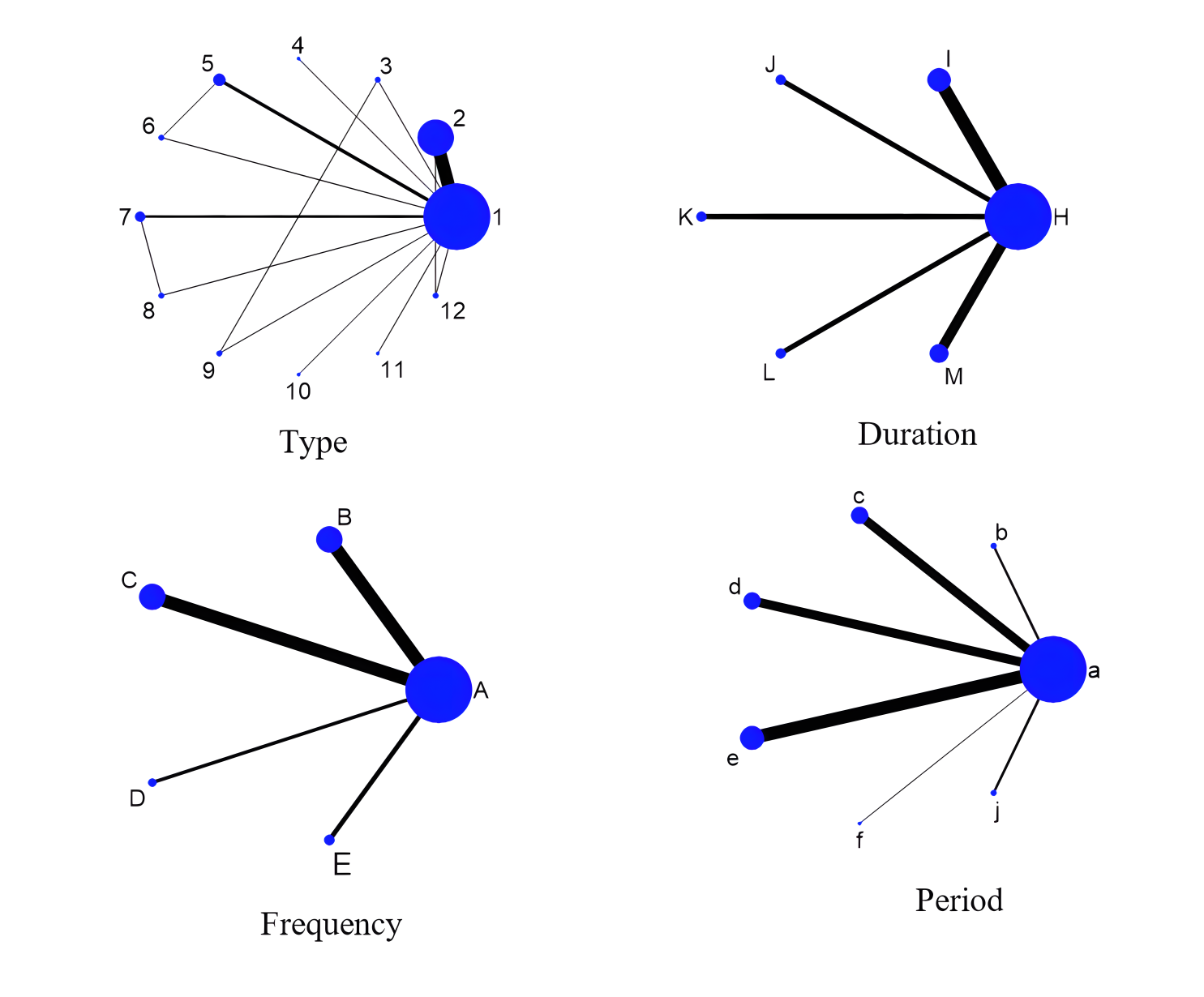


Figure 4 Network Plot of the Impact of Exercise Prescription Elements on Chronic Low Back

Pain Control in Adults

Note：1,con;2,Core stablization exercise;3,Combination exercise;4,Pilates;5,Yoga;6,Qigong;7,.Sling exercise;8,Swiss ball;9,Strength exercise;10,Disturbance exercise;11,Abdominal crunch exerciseA,;12,Taichi; A,con;B,1-2times;C,3times;D,5times;E,7times;H,con;I,15-30min;J,40min;K,45min;L,50min;M,≥60min;a,con;b,4weeks;c,6weeks;d,8weeks;e,12weeks;f,13weeks;j,≥16weeks.

## 3.4.2 Supplementary Figures

Figure 5: League Table of Pairwise Comparisons of Intervention Effects for Different Elements of Exercise Types

Figure 6: League Table of Pairwise Comparisons of Intervention Effects for Different Elements of Exercise Duration

Figure 7: League Table of Pairwise Comparisons of Intervention Effects for Different Elements of Exercise Frequency

Figure 8: League Table of Pairwise Comparisons of Intervention Effects for Different Elements of Exercise Period

## Note: The red numbers are statistically significant.

## 3.4.3 Supplementary Figures


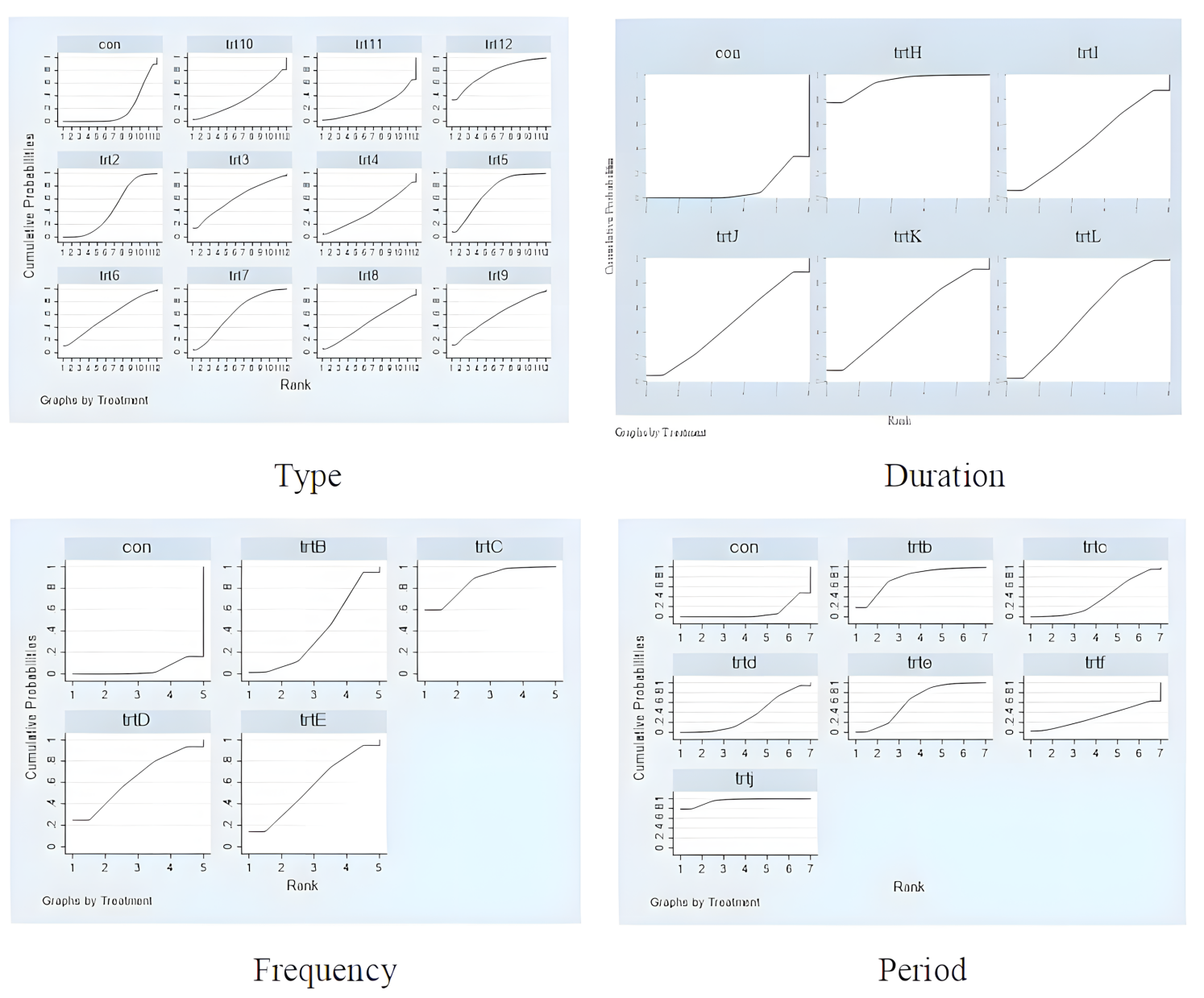


Figure 9: Probability Ranking Chart of Intervention Effects for Different Elements of Exercise Prescription Dose

Note:Type:con;trt2,Core stablization exercise;trt3,Combination exercise;trt4,Pilates;trt5,Yoga;trt6,Qigong;trt7,.Sling exercise;trt8,Swiss ball;trt9,Strength exercise;trt10Disturbance exercise;trt11,Abdominal crunch exercise;trt12,Taichi;Duration:con;trtH,15-30min;trtI,40min;trtJ,45min;trtK,45min;trtL,≥60min;Frequency:con;trtB,1-2times;trtC,3times;trtD,5times;trtE,7times;Period:con;trtb,4weeks;trtc,6weeks;trtd,8weeks;trte,12weeks;trtf,13weeks;trtj,≥16weeks.

## 3.4.5 Supplementary Figures


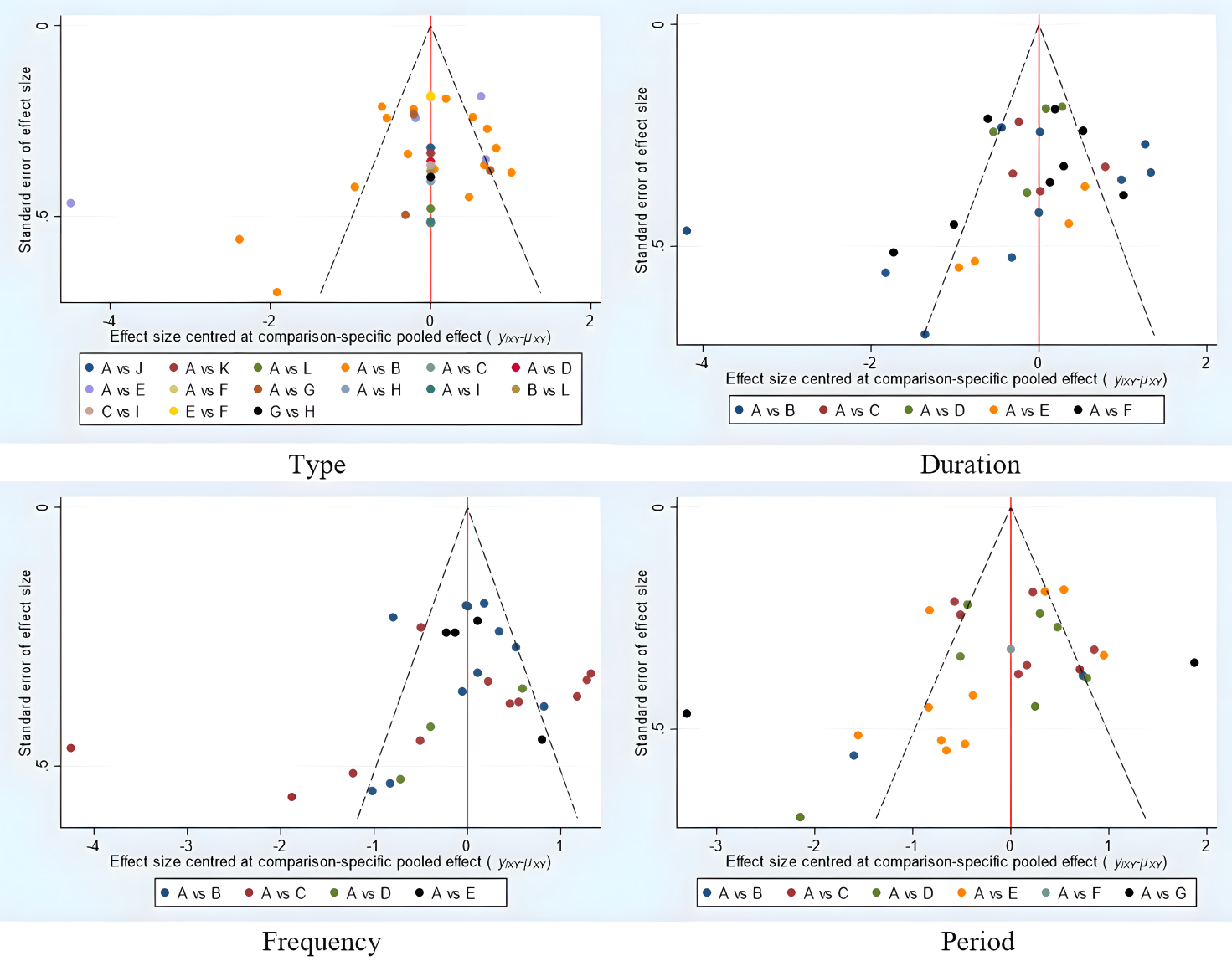


Figure 10: Funnel Plot of Intervention Effects for Different Elements of Exercise Prescription Dose

Note: Type: A, con; B, Core stablization exercise ;C, Combination exercise; D, Pilates; E, Yoga; F, Qigong; G, Sling exercise ; H ,Swiss ball ;I, Strength exercise ;J, Disturbance exercise ;K, Abdominal crunch exercise;L,Taichi;Duration:A,con;B,15-30min;C,40min;D,45min;E,50min,E,≥60min;Frequency:A,con;B,1-2times;C,3times;D,5times;E,7times;Period:A,con;B,4weeks;C,6weeks;D,8weeks;E,12weeks;F,13weeks;G≥16weeks.

## Supplementary Tables

Table 1 Evaluation of the quality of evidence in the included literature

| Author & Year | Limitations | Inconsistency | Indirectness | Imprecision | Publication bias | Level of Evidence |
| --- | --- | --- | --- | --- | --- | --- |
| Akhtar et al^(14)^，2017 | 0 | 0 | 0 | 0 | 0 | High |
| Arampatzis et al^(32)^，2017 | -1 | 0 | 0 | 0 | 0 | Moderate |
| Gladwell et al^(17)^，2006 | -1 | 0 | 0 | 0 | 0 | Moderate |
| Bae et al^(79)^，2018 | -1 | 0 | 0 | 0 | 0 | Moderate |
| Byoung-Hwan et al^(80)^，2015 | -1 | 0 | 0 | -1 | 0 | Low |
| Cho et al^(81)^，2015 | -1 | 0 | 0 | 0 | 0 | Moderate |
| Hwi-young et al^(82)^，2014 | -1 | 0 | -1 | 0 | 0 | Low |
| Hwangbo et al^(83)^，2015 | -1 | 0 | -1 | 0 | 0 | Low |
| Jing et al^(41)^，2019 | -1 | 0 | 0 | 0 | 0 | Moderate |
| Kimberly et al^(84)^，2005 | -1 | -1 | 0 | 0 | 0 | Low |
| Kumar et al^(12)^，2011 | 0 | 0 | 0 | 0 | 0 | High |
| Lee et al^(85)^，2016 | -1 | 0 | 0 | 0 | 0 | Moderate |
| Michaelson et al^(86)^，2016 | -1 | -1 | -1 | 0 | 0 | Very Low |
| Noormohammadpour et al^(87)^，2017 | -1 | -1 | 0 | 0 | 0 | Low |
| Roh et al^(88)^，2016 | -1 | 0 | 0 | 0 | 0 | Moderate |
| Shamsi et al^(15)^，2014 | -1 | 0 | 0 | 0 | 0 | Moderate |
| Tang et al^(18)^，2016 | -1 | 0 | 0 | 0 | 0 | Moderate |
| Tekur et al^(89)^，2012 | -1 | 0 | 0 | 0 | 0 | Moderate |
| Teut et al^(30)^，2016 | -1 | -1 | -1 | 0 | 0 | Very Low |
| Ulger et al^(67)^，2023 | -1 | 0 | 0 | 0 | 0 | Moderate |
| Ulger et al^(90)^，2017 | -1 | 0 | 0 | 0 | 0 | Moderate |
| Williams et al^(91)^，2009 | -1 | 0 | 0 | 0 | 0 | Moderate |
| Yoo et al^(57)^，2012 | -1 | 0 | 0 | 0 | 0 | Moderate |
| Zhang et al^(92)^，2015 | -1 | 0 | 0 | 0 | 0 | Moderate |
| Koumantakis et al^(13)^，2005 | -1 | 0 | 0 | 0 | 0 | Moderate |
| Joshua et al^(16)^，2013 | -1 | 0 | 0 | 0 | 0 | Moderate |

Table 2 Basic Characteristics of Included Literature

| Author & Year | Country | N | Mean age (years) | Instrument | Dose |
| --- | --- | --- | --- | --- | --- |
| Akhtar et al^(14)^，2017 | Pakistan | C=55 | 45.50±6.61 | Usual Care | 60min、2times、6weeks |
|  |  | E=53 | 46.39±7.43 | Core stabilization exercise |  |
| Arampatzis et al^(32)^，2017 | Germany | C=20 | 31.4±5.5 | Usual Care | 90min、2times、13weeks |
|  |  | E=20 | 31.9±6.0 | Disturbance exercise |  |
| Gladwell et al^(17)^，2006 | UK | C=14 | 45.9±8.0 | Usual Care | 60min、1time、6weeks |
|  |  | E=20 | 36.9±8.1 | Pilates |  |
| Bae et al^(79)^，2018 | Korea | C=18 | 32.4±10.7 | Abdominal crunch exercise | 30min、3times、12weeks |
|  |  | E=18 | 32.7±6.1 | Core stabilization exercise |  |
| Byoung-Hwan et al^(80)^，2015 | Korea | C=10 | 44.2±2.70 | Usual Care | 30min、5times、12weeks |
|  |  | E1=10 | 46.0±3.37 | Swiss ball |  |
|  |  | E2=10 | 46.2±3.22 | Sling exercise |  |
| Cho et al^(81)^，2015 | Korea | C=15 | 44.0±6.7 | Usual Care | 40min、3times、6weeks |
|  |  | E=15 | 48.1±6.9 | Core stabilization exercise |  |
| Hwi-young et al^(82)^，2014 | Korea | C=15 | 36.5±7.7 | Usual Care | 40min、3times、4weeks |
|  |  | E=15 | 38.1±7.9 | Core stabilization exercise |  |
| Hwangbo et al^(83)^，2015 | Korea | C=15 | 34.0±2.9 | Usual Care | 50min、3times、6weeks |
|  |  | E=15 | 34.5±4.0 | Core stabilization exercise |  |
| Jing et al^(41)^，2019 | Chinese | C=13 | 60.67±2.58 | Usual Care | 60min、3times、12weeks |
|  |  | E1=15 | 58.13±5.38 | Tai Chi |  |
|  |  | E2=15 | 58.4±5.08 | Core stabilization exercise |  |
| Kimberly et al^(84)^，2005 | Greece | C=26 | 35.2±9.7 | Usual Care | 50min、2times、8weeks |
|  |  | E=29 | 39.2.±11.4 | Core stabilization exercise |  |
| Kumar et al^(12)^，2011 | India | C=9 | 22.5±1.09 | Usual Care | 15min、8weeks |
|  |  | E=9 |  | Core stabilization exercise |  |
| Lee et al^(85)^，2016 | Korea | C=6 | 43.3±9.9 | Usual Care | 50min、2times、12weeks |
|  |  | E1=15 | 42.7±13.4 | Strength exercise |  |
|  |  | E2=15 | 46.7±8.1 | Combination exercise |  |
| Michaelson et al^(86)^，2016 | Sweden | C=35 | 52.1±17.16 | Usual Care | 120min、1time、8weeks |
|  |  | E=35 | 49.3±14.0 | Core stabilization exercise |  |
| Noormohammadpour et al^(87)^，2017 | Iran | C=10 | 41.3±6.4 | Usual Care | 8weeks |
|  |  | E=10 | 43.3±7.5 | Core stabilization exercise |  |
| Roh et al^(88)^，2016 | Korea | C=49 | 50.5±9.1 | Usual Care | 30min、3times、12weeks |
|  |  | E=53 | 49.5±10.6 | Sling exercise |  |
| Shamsi et al^(15)^，2014 | Iran | C=20 | 38.5±11.9 | Usual Care | 40min、3times、6weeks |
|  |  | E=19 | 47.7±10.4 | Core stabilization exercise |  |
| Tang et al^(18)^，2016 | Chinese | C=41 | 43.6±6.4 | Usual Care | 30min、7times、6weeks |
|  |  | E=41 | 41.7±5.6 | Core stabilization exercise |  |
| Tekur et al^(89)^，2012 | India | C=40 | 48.0±4.0 | Usual Care | 45min、7times、4weeks |
|  |  | E=40 | 49.0±3.6 | Core stabilization exercise |  |
| Teut et al^(30)^，2016 | Germany | C=57 | 72.6±6.0 | Usual Care | 45min、2times、12weeks |
|  |  | E1=61 | 73.0±5.6 | Yoga |  |
|  |  | E2=58 | 72.4±5.7 | Qigong |  |
| Ulger et al^(67)^，2023 | Turkey | C=12 | 55.08±2.67 | Core stabilization exercise | 60min、2times、8weeks |
|  |  | E=16 | 47.12±7.07 | Yoga |  |
| Ulger et al^(90)^，2017 | Turkey | C=56 | 41.6±12.9 | Usual Care | 60min、3times、6weeks |
|  |  | E=57 | 48.4±1.86 | Core stabilization exercise |  |
| Williams et al^(91)^，2009 | USA | C=47 | 47.6±1.47 | Usual Care | 30min、7times、24weeks |
|  |  | E=43 | 48.4±1.86 | Yoga |  |
| Yoo et al^(57)^，2012 | Korea | C=15 | 20.5±0.5 | Usual Care | 45min、3times、4weeks |
|  |  | E=15 | 20.1±0.7 | Sling exercise |  |
| Zhang et al^(92)^，2015 | Chinese | C=46 | 51.62±4.03 | Usual Care | 40min、7times、8weeks |
|  |  | E=46 | 48.71±3.8 | Core stabilization exercise |  |
| Koumantakis et al^(13)^，2005 | Greece | C=26 | 35.2±9.7 | Usual Care | 40-60min、2times、8weeks |
|  |  | E=29 | 39.2±11.4 | Core stabilization exercise |  |
| Joshua et al^(16)^，2013 | Korea | C=20 | 51.30±7.01 | Usual Care | 40min、3times、8weeks |
|  |  | E=20 | 50.35±9.26 | Core stabilization exercise |  |

Note: E=Experimental Group; C=Control Group.

Table 3 Risk of bias assessment of included studies (n=26) examining the efficacy of exercise training in patients with nonspecific chronic low back pain

| Study | Random  sequence  generation  (selection bias) | Allocation  concealment  (selection bias) | Blinding of  patients and  personnel  (performancebias) | Blinding of  outcome  assessment  (detection bias) | Incomplete  outcome data  (attrition bias) | Selective  outcome  reporting  (reporting  bias) | Any other bias |
| --- | --- | --- | --- | --- | --- | --- | --- |
| Akhtar et al^(14)^，2017 | High | Low | High | Unclear | High | Unclear | Low |
| Arampatzis et al^(32)^，2017 | Low | High | High | Low | Low | Low | Low |
| Gladwell et al^(17)^，2006 | High | High | Unclear | Unclear | Low | Low | Low |
| Bae et al^(79)^，2018 | Low | High | Unclear | High | Low | Low | Low |
| Byoung-Hwan et al^(80)^，2015 | High | Low | High | High | Low | Low | Low |
| Cho et al^(81)^，2015 | High | Low | High | Unclear | Low | Low | Low |
| Hwi-young et al^(82)^，2014 | Low | Unclear | Unclear | Unclear | Low | High | High |
| Hwangbo et al^(83)^，2015 | High | High | High | Low | Low | Low | Low |
| Jing et al^(41)^，2019 | Low | Low | High | Unclear | Low | Low | Low |
| Kimberly et al^(84)^，2005 | Unclear | High | High | Unclear | Low | Low | Low |
| Kumar et al^(12)^，2011 | Low | High | Low | Low | Low | Unclear | Low |
| Lee et al^(85)^，2016 | Low | Low | High | High | Low | Low | Low |
| Michaelson et al^(86)^，2016 | High | High | Unclear | Unclear | High | Low | Low |
| Noormohammadpour et al^(87)^，2017 | Low | High | High | Unclear | Low | Low | Low |
| Roh et al^(88)^，2016 | Low | Unclear | Unclear | Unclear | High | Unclear | High |
| Shamsi et al^(15)^，2014 | High | High | High | Low | Low | Low | Low |
| Tang et al^(18)^，2016 | Low | Unclear | Unclear | High | High | Unclear | Low |
| Tekur et al^(89)^，2012 | Low | Unclear | Unclear | Unclear | Low | Low | Low |
| Teut et al^(30)^，2016 | High | High | High | Unclear | High | Low | Low |
| Ulger et al^(67)^，2023 | Low | Low | High | High | Low | Unclear | Low |
| Ulger et al^(90)^，2017 | Unclear | High | High | Unclear | Low | Low | Low |
| Williams et al^(91)^，2009 | Low | Unclear | High | High | Unclear | High | Low |
| Yoo et al^(57)^，2012 | High | High | High | Unclear | Low | Unclear | Low |
| Zhang et al^(92)^，2015 | Low | Unclear | High | Unclear | High | Low | Low |
| Koumantakis et al^(13)^，2005 | Low | Unclear | Low | Unclear | High | Low | High |
| Joshua et al^(16)^，2013 | High | Low | Unclear | Unclear | Low | Unclear | Low |

Table 4 SUCRA Values for the Effectiveness of Interventions by Exercise Prescription Elements

| Rank | Type | SUCRA | Frequency | SUCRA | Duration | SUCRA | Period | SUCRA |
| --- | --- | --- | --- | --- | --- | --- | --- | --- |
| 1 | Taichi | 77.4 | ≥16weeks | 95.4 | 15-30min | 94.6 | 3times | 87.0 |
| 2 | Yoga | 72.1 | 4weeks | 78.1 | ≥60min | 55.9 | 5times | 63.6 |
| 3 | Sling exercise | 63.0 | 12weeks | 62.7 | 50min | 51.9 | 7times | 56.7 |
| 4 | Combination exercise | 61.6 | 6weeks | 37.4 | 45min | 47.7 | 1-2times | 38.4 |
| 5 | Strength exercise | 59.2 | 8weeks | 36.2 | 40min | 42.5 | con | 4.4 |
| 6 | Qigong | 57.5 | 13weeks | 31.1 | con | 7.3 |  |  |
| 7 | Swiss ball | 48.0 | con | 9.1 |  |  |  |  |
| 8 | Core stablization exercise | 44.8 |  |  |  |  |  |  |
| 9 | Pilates | 40.5 |  |  |  |  |  |  |
| 10 | Disturbance exercise | 34.5 |  |  |  |  |  |  |
| 11 | Abdominal crunch exercise | 23.4 |  |  |  |  |  |  |
| 12 | Con | 18.0 |  |  |  |  |  |  |

Table 5 Rational of excluding studies during the full-text screening

| No | Title | Reason for exclusion |
| --- | --- | --- |
| 1 | Impact of a 12-week Pilates-based exercise program on individuals with chronic low back pain: A randomized controlled trial | No available outcome |
| 2 | The effects of cognitive-behavioral therapy combined with physical exercise on chronic low back pain | No available outcome |
| 3 | Aerobic exercise versus resistance training for reducing pain and disability in individuals with chronic low back pain: A randomized controlled trial | No available outcome |
| 4 | Comparison of manual therapy and exercise therapy for chronic low back pain: A randomized controlled trial | No available outcome |
| 5 | Physical and emotional factors associated with the severity of chronic back pain in adults | No available outcome |
| 6 | Physical and emotional functioning of adult patients with chronic abdominal pain: Comparison with patients with chronic back pain | Non-available randomised controlled trial |
| 7 | Effect of aerobic exercise on pain, function, and quality of life in patients with chronic low back pain: A randomized controlled trial | Non-available randomised controlled trial |
| 8 | The effects of aquatic exercise on pain and mobility in individuals with chronic low back pain: A randomized controlled trial | Non-available randomised controlled trial |
| 9 | The effectiveness of strength training for the treatment of chronic low back pain: A systematic review and randomized controlled trial | Non-available randomised controlled trial |
| 10 | Efficacy of Tai Chi for the management of chronic low back pain in older adults: A randomized controlled trial | Non-available randomised controlled trial |
| 11 | Fiabilidad y validez de un programa de ejercicio para el tratamiento del dolor lumbar crónico: Un ensayo controlado aleatorizado | Non-English |
| 12 | Comparison of the effects of exercise therapy and psychological interventions on chronic low back pain: A randomized controlled trial | Duplicate reports for same trial |
| 13 | Impact of core stability exercise on chronic low back pain: A randomized controlled trial | Duplicate reports for same trial |
| 14 | The effect of a home-based exercise program for chronic low back pain: A randomized controlled trial | Duplicate reports for same trial |
| 15 | The effect of stretching exercises on pain intensity and quality of life in patients with chronic low back pain: A randomized controlled trial | Duplicate reports for same trial |
| 16 | A mind-body program for older adults with chronic low back pain: a randomized clinical trial | Duplicate reports for same trial |
| 17 | Exercise therapy versus traditional physiotherapy for chronic low back pain: A randomized controlled trial | Duplicate reports for same trial |
| 18 | Effects of whole-body electromyostimulation on chronic nonspecific low back pain in adults: a randomized controlled study | Duplicate reports for same trial |
| 19 | Efficacy of combined aerobic and strengthening exercises for chronic low back pain: A randomized controlled trial | Duplicate reports for same trial |
| 20 | Social inequalities in the prevalence of chronic back pain among adults in Germany | Irrelevant participants |
| 21 | Effects of high-intensity interval training on chronic low back pain in adults: A randomized controlled trial | Irrelevant participants |
| 22 | Efficacy of mindfulness-based stress reduction combined with physical exercise for chronic low back pain: A randomized controlled trial | Irrelevant participants |
| 23 | Conservative treatment of acute and chronic nonspecific low back pain: a systematic review of randomized controlled trials of the most common interventions | Irrelevant participants |
| 24 | Qigong or yoga versus no intervention in older adults with chronic low back pain—a randomized controlled trial | Irrelevant participants |
| 25 | Effects of a 6-month exercise program on pain and function in individuals with chronic low back pain: A randomized controlled trial | Irrelevant participants |
| 26 | The impact of a combined aerobic and resistance exercise program on chronic low back pain: A randomized controlled trial | Irrelevant participants |
| 27 | The efficacy of home-based stretching exercises for chronic low back pain: A randomized controlled trial | Irrelevant participants |
| 28 | Cigarette smoking and chronic low back pain in the adult population | Irrelevant participants |
| 29 | Virtual reality exergame for supplementing multimodal pain therapy in older adults with chronic back pain: a randomized controlled pilot study | Irrelevant participants |
| 30 | The prevalence of low back pain in adults: a methodological review of the literature | Irrelevant participants |
| 31 | Feasibility and long-term efficacy of a proactive health program in the treatment of chronic back pain: a randomized controlled trial | Irrelevant participants |
| 32 | A prospective, double-blind, pilot, randomized, controlled trial of an “embodied” virtual reality intervention for adults with low back pain | Irrelevant participants |
| 33 | A randomized controlled trial of intensive neurophysiology education in chronic low back pain | Irrelevant participants |
| 34 | Outcome comparison among working adults with centralizing low back pain: secondary analysis of a randomized controlled trial with 1-year follow-up | Irrelevant participants |
| 35 | The clinical course of low back pain: a meta-analysis comparing outcomes in randomised clinical trials (RCTs) and observational studies | Missing data |
| 36 | Effects of open-label placebo on pain, functional disability, and spine mobility in patients with chronic back pain: a randomized controlled trial | Missing data |
| 37 | Yoga vs. conventional physiotherapy for treating chronic low back pain in adults: A randomized controlled trial | Missing data |
| 38 | Comparing yoga, exercise, and a self-care book for chronic low back pain: a randomized, controlled trial | Defects in statistical methods |
| 39 | A comprehensive yoga programs improves pain, anxiety and depression in chronic low back pain patients more than exercise: an RCT | Defects in statistical methods |
| 40 | Effect of progressive postural control exercise versus core stability exercise in young adults with chronic low back pain: A randomized controlled trial | Defects in statistical methods |
| 41 | A comparison of strength training and aerobic exercise for the management of chronic low back pain | Defects in statistical methods |
| 42 | Efficacy of supervised exercise therapy in treating adults with non-specific chronic low back pain: A randomized controlled trial | Defects in statistical methods |
| 43 | Continuous low-level heat wrap therapy for the prevention and early phase treatment of delayed-onset muscle soreness of the low back: A randomized controlled trial | Meeting |
| 44 | Physiotherapy for Sleep Disturbance in People With Chronic Low Back Pain: Results of a Feasibility Randomized Controlled Trial | Meeting |
| 45 | Open-label placebo treatment in chronic low back pain: a randomized controlled trial | Meeting |
| 46 | Communication Skills Training for Practitioners to. Increase Patient Adherence to Home-Based Rehabilitation for Chronic Low Back Pain: Results of a Cluster Randomized Controlled Trial | Meeting |
| 47 | Video-Game-Based Exercises for Older People With Chronic Low Back Pain: A Randomized Controlledtable Trial | Meeting |
| 48 | Effect of a Self-Determination Theory-Based Communication Skills Training Program on Physiotherapists' Psychological Support for Their Patients With Chronic Low Back Pain: A Randomized Controlled Trial | Meeting |
| 49 | Opioids versus physical therapy for management of chronic back pain | Irrelevant interventions or comparisons |
| 50 | Randomised controlled trial of integrated care to reduce disability from chronic low back pain in working and private life | Irrelevant interventions or comparisons |
| 51 | Effects of exercise intervention on chronic low back pain in adults: A randomized controlled trial | Irrelevant interventions or comparisons |
| 52 | The impact of aerobic exercise on pain intensity and functional disability in adults with chronic low back pain: A randomized controlled trial | Irrelevant interventions or comparisons |
| 53 | Comparing the effectiveness of strength training and aerobic exercise for adults with chronic low back pain: A randomized controlled trial | Irrelevant interventions or comparisons |
| 54 | A randomized controlled trial of yoga and physical therapy for managing chronic low back pain in adults | Irrelevant interventions or comparisons |
| 55 | Effectiveness of a combined exercise program for chronic low back pain management: A randomized controlled trial | Irrelevant interventions or comparisons |
| 56 | The effects of Pilates-based exercise on chronic low back pain: A randomized controlled trial | Irrelevant interventions or comparisons |
| 57 | Effect of a physical therapy intervention on chronic low back pain: A multicenter randomized controlled trial. | Irrelevant interventions or comparisons |
| 58 | A randomized controlled trial of stretching exercises for managing chronic low back pain in adults | Irrelevant interventions or comparisons |
| 59 | Comparing the efficacy of yoga and conventional exercise for chronic low back pain in adults: A randomized controlled trial | Irrelevant interventions or comparisons |
| 60 | The impact of strength and flexibility training on chronic low back pain in adults: A randomized controlled trial | Irrelevant interventions or comparisons |
| 61 | Effectiveness of aquatic exercise for chronic low back pain: A randomized controlled trial | Irrelevant interventions or comparisons |
| 62 | The effects of cognitive behavioral therapy combined with exercise on chronic low back pain: A randomized controlled trial | Irrelevant interventions or comparisons |
| 63 | Effectiveness of aerobic exercise for reducing chronic low back pain: A randomized controlled trial | Irrelevant interventions or comparisons |
| 64 | A randomized controlled trial of resistance training for chronic low back pain management | Irrelevant interventions or comparisons |
| 65 | The efficacy of tai chi in managing chronic low back pain: A randomized controlled trial | Irrelevant interventions or comparisons |
| 66 | Effect of combined exercise interventions on chronic low back pain in adults: A randomized controlled trial | Irrelevant interventions or comparisons |
| 67 | A randomized controlled trial comparing the effectiveness of physical therapy and home-based exercise for chronic low back pain | Irrelevant interventions or comparisons |
| 68 | The effect of combined aerobic and strength training on chronic low back pain: A randomized controlled trial | Irrelevant interventions or comparisons |
| 69 | The effects of Pilates exercise on pain relief and functional improvement in chronic low back pain: A randomized controlled trial | Irrelevant interventions or comparisons |
| 70 | Evaluating the impact of strength and flexibility exercise on chronic low back pain: A randomized controlled trial | Irrelevant interventions or comparisons |
| 71 | Yoga for chronic low back pain: A randomized controlled trial | Irrelevant interventions or comparisons |
| 72 | The effectiveness of physical exercise interventions for chronic low back pain: A randomized controlled trial | Irrelevant interventions or comparisons |
| 73 | A randomized controlled trial of Pilates exercise for patients with chronic low back pain: Effects on pain, function, and quality of life | Irrelevant interventions or comparisons |
| 74 | Effects of a yoga-based program on pain reduction and functional improvement in chronic low back pain: A randomized controlled trial | Irrelevant interventions or comparisons |
| 75 | The effect of a mindfulness-based intervention combined with physical exercise on chronic low back pain: A randomized controlled trial | Irrelevant interventions or comparisons |
| 76 | The effectiveness of high-intensity interval training for managing chronic low back pain in adults: A randomized controlled trial | Irrelevant interventions or comparisons |
| 77 | A randomized controlled trial on the effectiveness of progressive resistance training for chronic low back pain management | Irrelevant interventions or comparisons |
| 78 | The role of combined aerobic exercise and education in chronic low back pain management: A randomized controlled trial | Irrelevant interventions or comparisons |
| 79 | The effects of multidisciplinary rehabilitation programs on chronic low back pain: A randomized controlled trial | Irrelevant interventions or comparisons |
| 80 | Effectiveness of an interdisciplinary rehabilitation program for chronic low back pain in adults: A randomized controlled trial | Irrelevant interventions or comparisons |
| 81 | A randomized controlled trial of the effects of Tai Chi on chronic low back pain: A comparison of group-based versus home-based intervention | Irrelevant interventions or comparisons |
| 82 | The impact of a stretching and strengthening program on chronic low back pain: A randomized controlled trial | Irrelevant interventions or comparisons |
| 83 | The effects of supervised exercise training on pain and physical function in individuals with chronic low back pain: A randomized controlled trial | Irrelevant interventions or comparisons |
| 84 | A comparison of Pilates and standard physical therapy for the treatment of chronic low back pain: A randomized controlled trial | Irrelevant interventions or comparisons |

Table 6 PRISMA 2020 Checklist

| **Section and Topic** | **Item #** | **Checklist item** | **Location where item is reported** |
| --- | --- | --- | --- |
| **TITLE** | | |  |
| Title | 1 | Identify the report as a systematic review. | Page 1 |
| **ABSTRACT** | | |  |
| Abstract | 2 | See the PRISMA 2020 for Abstracts checklist. | Page 1 |
| **INTRODUCTION** | | |  |
| Rationale | 3 | Describe the rationale for the review in the context of existing knowledge. | Page 2 |
| Objectives | 4 | Provide an explicit statement of the objective(s) or question(s) the review addresses. | Page 2 |
| **METHODS** | | |  |
| Eligibility criteria | 5 | Specify the inclusion and exclusion criteria for the review and how studies were grouped for the syntheses. | Page 3 |
| Information sources | 6 | Specify all databases, registers, websites, organisations, reference lists and other sources searched or consulted to identify studies. Specify the date when each source was last searched or consulted. | Page 2 |
| Search strategy | 7 | Present the full search strategies for all databases, registers and websites, including any filters and limits used. | Page 2-3 |
| Selection process | 8 | Specify the methods used to decide whether a study met the inclusion criteria of the review, including how many reviewers screened each record and each report retrieved, whether they worked independently, and if applicable, details of automation tools used in the process. | Page 3-4 |
| Data collection process | 9 | Specify the methods used to collect data from reports, including how many reviewers collected data from each report, whether they worked independently, any processes for obtaining or confirming data from study investigators, and if applicable, details of automation tools used in the process. | Page 4 |
| Data items | 10a | List and define all outcomes for which data were sought. Specify whether all results that were compatible with each outcome domain in each study were sought (e.g. for all measures, time points, analyses), and if not, the methods used to decide which results to collect. | Page 3 |
|  | 10b | List and define all other variables for which data were sought (e.g. participant and intervention characteristics, funding sources). Describe any assumptions made about any missing or unclear information. | Page 3 |
| Study risk of bias assessment | 11 | Specify the methods used to assess risk of bias in the included studies, including details of the tool(s) used, how many reviewers assessed each study and whether they worked independently, and if applicable, details of automation tools used in the process. | Page 5 |
| Effect measures | 12 | Specify for each outcome the effect measure(s) (e.g. risk ratio, mean difference) used in the synthesis or presentation of results. | Page 3 |
| Synthesis methods | 13a | Describe the processes used to decide which studies were eligible for each synthesis (e.g. tabulating the study intervention characteristics and comparing against the planned groups for each synthesis (item #5)). | Page 3 |
|  | 13b | Describe any methods required to prepare the data for presentation or synthesis, such as handling of missing summary statistics, or data conversions. | Page 4 |
|  | 13c | Describe any methods used to tabulate or visually display results of individual studies and syntheses. | Page 4 |
|  | 13d | Describe any methods used to synthesize results and provide a rationale for the choice(s). If meta-analysis was performed, describe the model(s), method(s) to identify the presence and extent of statistical heterogeneity, and software package(s) used. | Page 4 |
|  | 13e | Describe any methods used to explore possible causes of heterogeneity among study results (e.g. subgroup analysis, meta-regression). | Page 3 |
|  | 13f | Describe any sensitivity analyses conducted to assess robustness of the synthesized results. | Page 6 |
| Reporting bias assessment | 14 | Describe any methods used to assess risk of bias due to missing results in a synthesis (arising from reporting biases). | Page 6 |
| Certainty assessment | 15 | Describe any methods used to assess certainty (or confidence) in the body of evidence for an outcome. | Page 6 |
| **RESULTS** | | |  |
| Study selection | 16a | Describe the results of the search and selection process, from the number of records identified in the search to the number of studies included in the review, ideally using a flow diagram. | Page 19 |
|  | 16b | Cite studies that might appear to meet the inclusion criteria, but which were excluded, and explain why they were excluded. | Page 3 |
| Study characteristics | 17 | Cite each included study and present its characteristics. | Page 27-28 |
| Risk of bias in studies | 18 | Present assessments of risk of bias for each included study. | Page 20-21 |
| Results of individual studies | 19 | For all outcomes, present, for each study: (a) summary statistics for each group (where appropriate) and (b) an effect estimate and its precision (e.g. confidence/credible interval), ideally using structured tables or plots. | Page 5 |
| Results of syntheses | 20a | For each synthesis, briefly summarise the characteristics and risk of bias among contributing studies. | Page 6 |
|  | 20b | Present results of all statistical syntheses conducted. If meta-analysis was done, present for each the summary estimate and its precision (e.g. confidence/credible interval) and measures of statistical heterogeneity. If comparing groups, describe the direction of the effect. | N/A |
|  | 20c | Present results of all investigations of possible causes of heterogeneity among study results. | Page 5 |
|  | 20d | Present results of all sensitivity analyses conducted to assess the robustness of the synthesized results. | Page 4 |
| Reporting biases | 21 | Present assessments of risk of bias due to missing results (arising from reporting biases) for each synthesis assessed. | Page 6 |
| Certainty of evidence | 22 | Present assessments of certainty (or confidence) in the body of evidence for each outcome assessed. | Page 6 |
| **DISCUSSION** | | |  |
| Discussion | 23a | Provide a general interpretation of the results in the context of other evidence. | Page 10 |
|  | 23b | Discuss any limitations of the evidence included in the review. | Page 9 |
|  | 23c | Discuss any limitations of the review processes used. | Page 9 |
|  | 23d | Discuss implications of the results for practice, policy, and future research. | Page 9 |
| **OTHER INFORMATION** | | |  |
| Registration and protocol | 24a | Provide registration information for the review, including register name and registration number, or state that the review was not registered. | Page 2 |
|  | 24b | Indicate where the review protocol can be accessed, or state that a protocol was not prepared. | Page 2 |
|  | 24c | Describe and explain any amendments to information provided at registration or in the protocol. | Page 2 |
| Support | 25 | Describe sources of financial or non-financial support for the review, and the role of the funders or sponsors in the review. | Page 10 |
| Competing interests | 26 | Declare any competing interests of review authors. | Page 10 |
| Availability of data, code and other materials | 27 | Report which of the following are publicly available and where they can be found: template data collection forms; data extracted from included studies; data used for all analyses; analytic code; any other materials used in the review. | Page 2-3 |
